# METTL8 links mt-tRNA m^3^C modification to the HIF1α/RTK/Akt axis to sustain GBM stemness and tumorigenicity

**DOI:** 10.1038/s41419-024-06718-2

**Published:** 2024-05-14

**Authors:** Bernice Woon Li Lee, You Heng Chuah, Jeehyun Yoon, Oleg V. Grinchuk, Yajing Liang, Jayshree L. Hirpara, Yating Shen, Loo Chien Wang, Yan Ting Lim, Tianyun Zhao, Radoslaw M. Sobota, Tseng Tsai Yeo, Andrea Li Ann Wong, Kejia Teo, Vincent Diong Weng Nga, Bryce Wei Quan Tan, Toshio Suda, Tan Boon Toh, Shazib Pervaiz, Zhewang Lin, Derrick Sek Tong Ong

**Affiliations:** 1 Department of Physiology, Yong Loo Lin School of Medicine, National University of Singapore, Singapore, 117593 Singapore; 2 NUS Center for Cancer Research, Yong Loo Lin School of Medicine, National University of Singapore, Singapore, Singapore; 3 Cancer Science Institute of Singapore, National University of Singapore, Singapore, 117599 Singapore; 4 The N.1 Institute for Health, National University of Singapore, Singapore, Singapore; 5 The Institute for Digital Medicine (WisDM), Yong Loo Lin School of Medicine, National University of Singapore, Singapore, Singapore; 6 Functional Proteomics Laboratory, SingMass National Laboratory, Institute of Molecular and Cell Biology, Agency for Science, Technology and Research (A*STAR), Singapore, Singapore; 7 Department of Surgery, Division of Neurosurgery, National University Hospital, Singapore, Singapore; 8 Department of Haematology-Oncology, National University Hospital, Singapore, Singapore; 9 Department of Medicine, National University Hospital, Singapore, Singapore; 10 International Research Center for Medical Sciences, Kumamoto University, Kumamoto, 860-0811 Japan; 11 Healthy Longevity Translational Research Programme, Yong Loo Lin School of Medicine, National University of Singapore, Singapore, Singapore; 12 Department of Biological Sciences, 14 Science Drive 4, National University of Singapore, 117543 Singapore, Singapore; 13 Institute of Molecular and Cell Biology (IMCB), Agency for Science, Technology and Research (A*STAR), Singapore, Singapore; 14 National Neuroscience Institute, 308433 Singapore, Singapore

**Keywords:** CNS cancer, RNA modification

## Abstract

Epitranscriptomic RNA modifications are crucial for the maintenance of glioma stem cells (GSCs), the most malignant cells in glioblastoma (GBM). 3-methylcytosine (m^3^C) is a new epitranscriptomic mark on RNAs and METTL8 represents an m^3^C writer that is dysregulated in cancer. Although METTL8 has an established function in mitochondrial tRNA (mt-tRNA) m^3^C modification, alternative splicing of *METTL8* can also generate isoforms that localize to the nucleolus where they may regulate R-loop formation. The molecular basis for METTL8 dysregulation in GBM, and which METTL8 isoform(s) may influence GBM cell fate and malignancy remain elusive. Here, we investigated the role of METTL8 in regulating GBM stemness and tumorigenicity. In GSC, METTL8 is exclusively localized to the mitochondrial matrix where it installs m^3^C on mt-tRNA^Thr/Ser(UCN)^ for mitochondrial translation and respiration. High expression of *METTL8* in GBM is attributed to histone variant H2AZ-mediated chromatin accessibility of HIF1α and portends inferior glioma patient outcome. *METTL8* depletion impairs the ability of GSC to self-renew and differentiate, thus retarding tumor growth in an intracranial GBM xenograft model. Interestingly, *METTL8* depletion decreases protein levels of HIF1α, which serves as a transcription factor for several receptor tyrosine kinase (RTK) genes, in GSC. Accordingly, METTL8 loss inactivates the RTK/Akt axis leading to heightened sensitivity to Akt inhibitor treatment. These mechanistic findings, along with the intimate link between METTL8 levels and the HIF1α/RTK/Akt axis in glioma patients, guided us to propose a HIF1α/Akt inhibitor combination which potently compromises GSC proliferation/self-renewal in vitro. Thus, METTL8 represents a new GBM dependency that is therapeutically targetable.

## Introduction

Glioblastoma (GBM) is the most lethal form of primary brain tumors that has seen little progress in its clinical management, which remains to be aggressive surgery, radiation and chemo-therapies. A subset of GBM cells, commonly known as glioma stem cells (GSCs), imparts GBM with the ability to self-renew and differentiate, invade through the normal parenchyma, resist therapeutic insults and initiate tumor formation in immunocompromised mice [1–4]. The prevailing view is that GSC eradication would translate into therapeutic benefit, motivating the use of patient-derived GSCs to identify actionable biological insights that would expand our arsenal for GBM treatment [5]. We have previously employed patient-derived GSCs to uncover novel GBM dependencies on biotin distribution, H2AZ-mediated chromatin accessibility for cell cycle gene regulation, and suppression of RNF8-mediated mitotic checkpoint [6–8]. Importantly, our mechanistic studies have also yielded rational combination therapies (using existing pharmacologic agents) that indirectly target these GBM dependencies.

Epitranscriptomics is an emerging field in cancer biology and biomedicine, and includes reversible RNA modifications and ADAR-mediated adenosine-to-inosine RNA editing, which in turn affect RNA stability, translation efficiency, secondary structures, subcellular localization, alternative splicing and polyadenylation [9]. For instance, the m^6^A writer (METTL3), eraser (ALKBH5) and reader (YTHDF2) are overexpressed in GBM, where they regulate GSC self-renewal and tumorigenicity by reducing the mRNA stability of *OPTN* (thereby downregulating mitophagy) [10]; as well as stabilizing RNAs of GSC critical genes such as *FOXM1* and *MYC* [11, 12]. In addition, high expression of PUS7 facilitates pseudouridylation on tRNAs to inhibit mRNA translation of *TYK2*, hence attenuating IFN-STAT1 pathway in GSCs [13]. Interestingly, 3-methylcytosine (m^3^C) modifications on cytoplasmic and mitochondrial (mt) tRNAs have been recently reported but their role in GBM pathogenesis remain unexplored [14–17].

METTL8 is an m^3^C methyltransferase that is best known for its mitochondrial role in installing m^3^C_32_ on mt-tRNA^Thr/Ser(UCN)^ [14, 15]. In this way, METTL8 prevents mitoribosome stalling on mt-tRNA^Ser(UCN)^-dependent codons and promotes mt-tRNA^Thr/Ser(UCN)^ folding/ stability, thereby enhancing mitochondrial translation and respiration [14, 15]. Interestingly, *METTL8* has multiple splicing isoforms: isoform 1 being localized to the mitochondria while isoforms 3 and 4 (without the mitochondria-targeting signal) reside in the nucleolus [18]. The nucleolar METTL8 can undergo sumoylation and associate with nuclear RNA-binding proteins to regulate R-loop formation on ribosomal DNA gene (presumably via its methyltransferase activity on m^3^C) [19]. To complicate matters, there are also reports of METTL8 binding to mRNAs including *ARID1A* and *MAPKBP1*: the former leading to increased ARID1A protein levels that promote migration of breast cancer cells; while the latter results in the inhibition of MAPKBP1 translation thereby inhibiting the JNK pathway, which enhances mouse embryonic stem cell differentiation [20, 21]. Although *METTL8* is overexpressed in numerous cancer types [15], little is known about what contributes to aberrant *METTL8* expression in GBM and which METTL8 isoform(s) may influence GBM cell fate and malignancy. In this study, we explored the role of METTL8 in GBM proliferation, stemness and tumorigenicity; and investigated *METTL8* loss-associated molecular alterations that may allow us to identify pharmacologically actionable nodes to target GSC.

## Results

### *METTL8* overexpression in GBM is attributed to H2AZ-mediated chromatin accessibility of HIF1α

**Fig. 1:**
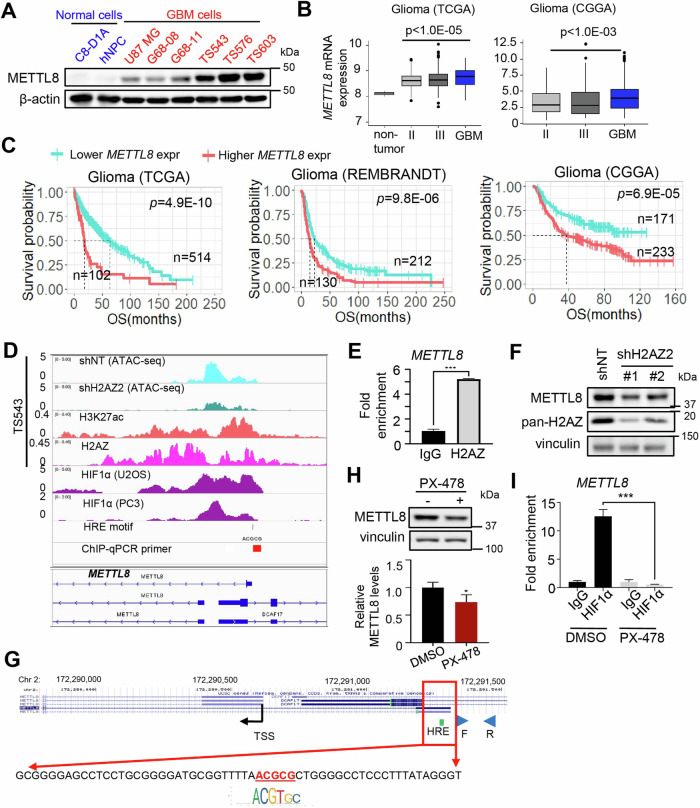
GSCs overexpress *METTL8* via H2AZ-mediated chromatin accessibility of HIF1α. **A** Western blot analysis of METTL8 levels in mouse astrocyte C8-D1A, human neural progenitor cells (NPC), U87 MG and patient-derived GSCs. **B** Comparison of *METTL8* mRNA levels in non-tumor tissues and gliomas of different clinical grades in TCGA and CGGA cohorts. Wilcoxon–Mann Whitney test. **C** Correlative analysis of *METTL8* levels with glioma patient survival in multiple glioma patient cohorts. OS overall survival. Wald test. **D** Integrative analysis of ATAC-Seq data (shH2AZ2 vs shNT), as well as H3K27ac, H2AZ and HIF1α ChIP-Seq data of *METTL8* proximal promoter, along with the location of ChIP-qPCR primers and predicted HRE motif. **E** ChIP-qPCR analysis of H2AZ occupancy on the *METTL8* promoter in GSC (*n* = 3) (mean ± SD), ***P < 0.001. **F** Western blot analysis of H2AZ and METTL8 protein levels in *H2AZ2* KD GSC. **G** Zoom-in view of the *METTL8* promoter to highlight the predicted HRE motif, along with the location of ChIP-qPCR primers. **H** Western blot analysis of METTL8 levels in GSC TS576 treated with or without 25 µM PX-478 for 1 day. The relative METTL8 levels was normalized to DMSO control (*n* = 3) (mean ± SD) *P < 0.05. **I** ChIP-qPCR analysis of HIF1α occupancy on the *METTL8* promoter upon PX-478 (25 µM, 1 day) treatment of GSC (*n* = 3) (mean ± SD), ***P < 0.001.

We first compared METTL8 expression in a panel of GBM cell lines vs non-cancerous brain cells, and validated METTL8 overexpression in GBM cells (Fig. 1A). In multiple patient cohorts, *METTL8* expression is also significantly higher in high-grade than low-grade gliomas (Fig. 1B). Using a published single-cell RNA-Seq dataset of human GBM, we further show that *METTL8* is heterogeneously expressed in GBM; with overall higher *METTL8* expression in GBM than non-GBM cells within the tumor microenvironment (Supplementary Fig. S1A). Crucially, higher *METTL8* levels correlate with inferior glioma patient outcome in multiple patient cohorts (Fig. 1C). Unexpectedly, we observed histone variant H2AZ enrichment at the *METTL8* gene promoter in GSC from our reported H2AZ ChIP-Seq analysis of GSC, concordant with the presence of H3K27ac mark (active enhancer) [8] (Fig. 1D). This observation was reinforced by stronger H3K27ac marks at the *METTL8* gene promoter in GBM tissues vs normal brain tissues (Supplementary Fig. S1B). Indeed, *H2AZ2* depletion reduces chromatin accessibility of *METTL8* gene promoter as revealed by ATAC-Seq analysis (Fig. 1D). We confirmed H2AZ enrichment at the *METTL8* gene promoter in GSC TS576 by using H2AZ ChIP-qPCR analysis, and *H2AZ2* depletion reduced METTL8 levels (Fig. 1E, F).

To explore transcriptional activators of *METTL8* in glioma, we conducted correlative analysis of *METTL8* expression with that of established GBM-relevant transcription factors in gliomas using TCGA, NCI REMBRANDT and Gravendeel datasets [22–26]. Only *HIF1A* and *STAT3* robustly showed significant positive correlations with *METTL8* in all three glioma cohorts (Supplementary Fig. S1C). Furthermore, the *METTL8* gene promoter harbored a putative HIF1α binding site based on publicly available HIF1α ChIP-Seq datasets and a predicted hypoxia-response element (HRE) motif (Fig. 1D, G). To determine if HIF1α can regulate *METTL8* transcription, we treated GSC TS576 with PX-478 (a HIF1α-specific inhibitor that suppresses HIF1α protein expression and transactivation activity under normoxic and hypoxic conditions [27]) for 1 day. This significantly decreased METTL8 levels, concomitant with decreased enrichment of HIF1α at the *METTL8* gene promoter (Fig. 1H, I). In contrast, neither E2F inhibitor (HLM006474) nor STAT3 inhibitor (S3I-201) treatment downregulated METTL8 levels in GSC TS576 (Supplementary Fig. S1D, E). Collectively, we show that GBM overexpresses *METTL8* via H2AZ-mediated chromatin accessibility of HIF1α.

### *METTL8* depletion impairs GSC stemness and tumorigenicity

**Fig. 2:**
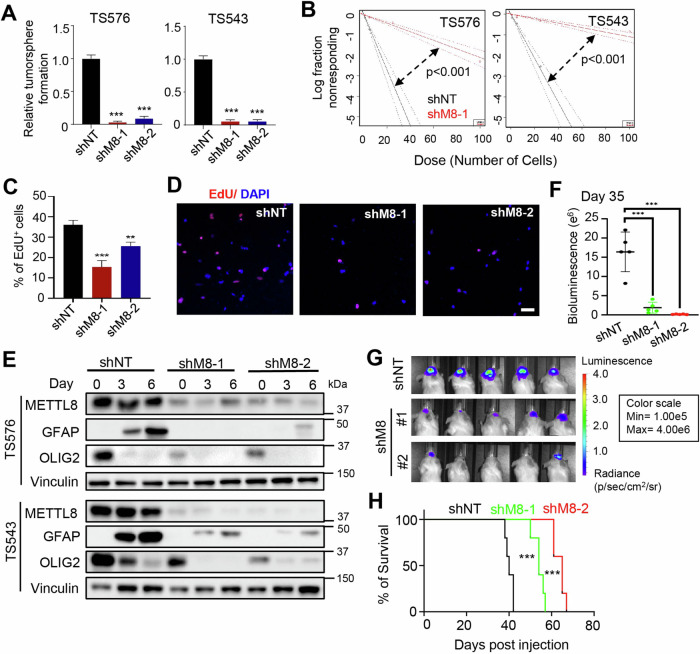
*METTL8* depletion impairs GSC stemness and tumorigenicity. **A** Tumorsphere formation of GSCs following *METTL8* KD (*n* = 6) (mean ± SD). ***P < 0.001. **B** In vitro limiting dilution assays of GSCs transduced with NT/control or METTL8 shRNA calculated with ELDA analysis. **C** Quantification of EdU^+^ cells upon *METTL8* KD (*n* = 3) (mean ± SD). Approximately 200 nuclei were counted per replicate. **P < 0.01, ***P < 0.001. **D** Representative images of EdU immunofluorescence. Scale bar: 50 µm. **E** Western blot analysis of GFAP and OLIG2 levels in *METTL8* KD GSC upon serum-induced differentiation. **F**, **G** In vivo bioluminescence-based imaging 35 days post-orthotropic injection of GSC TS576 (7.5 × 10^5^ cells) transduced with NT/control or METTL8 shRNAs. Quantification of tumor volume based on bioluminescence (**F**) and representative images of the tumor-bearing mice (**G**) (*n* = 5) (mean ± SD). ***P < 0.001. Two-tailed unpaired Student’s *t*-test. **H** Survival curves of mice implanted with GSC transduced with NT or METTL8 shRNAs. ***P < 0.001. Log-rank test.

To determine the role of METTL8 in GSC biology, we employed a variety of well-established in vitro and in vivo assays, including the tumorsphere assay (a readout for GSC proliferation/self-renewal); soft agar colony formation assay (a readout for GSC clonogenicity and transforming potential); extreme limiting dilution assay (a readout for tumor-initiating cell frequency); Transwell migration and invasion assay; and xenotransplantation assay [7, 28]. *METTL8* silencing led to a profound reduction in tumorsphere formation, colony formation and tumor initiating cell frequency of independent two GSC lines (Supplementary Fig. S2A–C; Fig. 2A, B). This aligned with a significant reduction in EdU^+^ GSC (in EdU transient labeling assay), as well as an increased apoptosis upon *METTL8* silencing (Fig. 2C, D; Supplementary Fig. 2A). Moreover, *METTL8* depletion downregulated OLIG2 (an established GSC marker) and compromised the ability of GSC to undergo serum-induced differentiation (as reported by GFAP levels, an astrocytic marker) when compared to the non-targeting shRNA control, indicating that METTL8 is essential for GSC stemness (Fig. 2E). *METTL8* depleted GSCs were also significantly less invasive than their *METTL8* intact counterpart (Supplementary Fig. 2D). In addition, we asked if METTL8 may influence GSC response to Temozolomide (TMZ), a DNA alkylating agent used as standard of care for GBM patients. Although the levels of γH2AX were similar among TMZ treated-GSCs regardless of METTL8 expression, there is an increase in apoptosis of *METTL8* depleted GSCs upon TMZ challenge when compared to the shNT control, suggesting that *METTL8* loss mitigates TMZ resistance of GSC (Supplementary Fig. 2E). In xenotransplantation experiments, the tumorigenic potential of GSC was significantly lessened upon *METTL8* depletion (Fig. 2F, G). Accordingly, mice bearing *METTL8* depleted GSCs survived significantly longer than those bearing *METTL8* intact GSCs (median survival of shM8-1 = 54 days and shM8-2 = 65 days vs shNT = 40 days) (Fig. 2H). We conclude that *METTL8* is crucial for GBM stemness and tumorigenicity.

### METTL8 mediates mt-tRNA m^3^C modification for mitochondrial translation and respiration in GSC

**Fig. 3:**
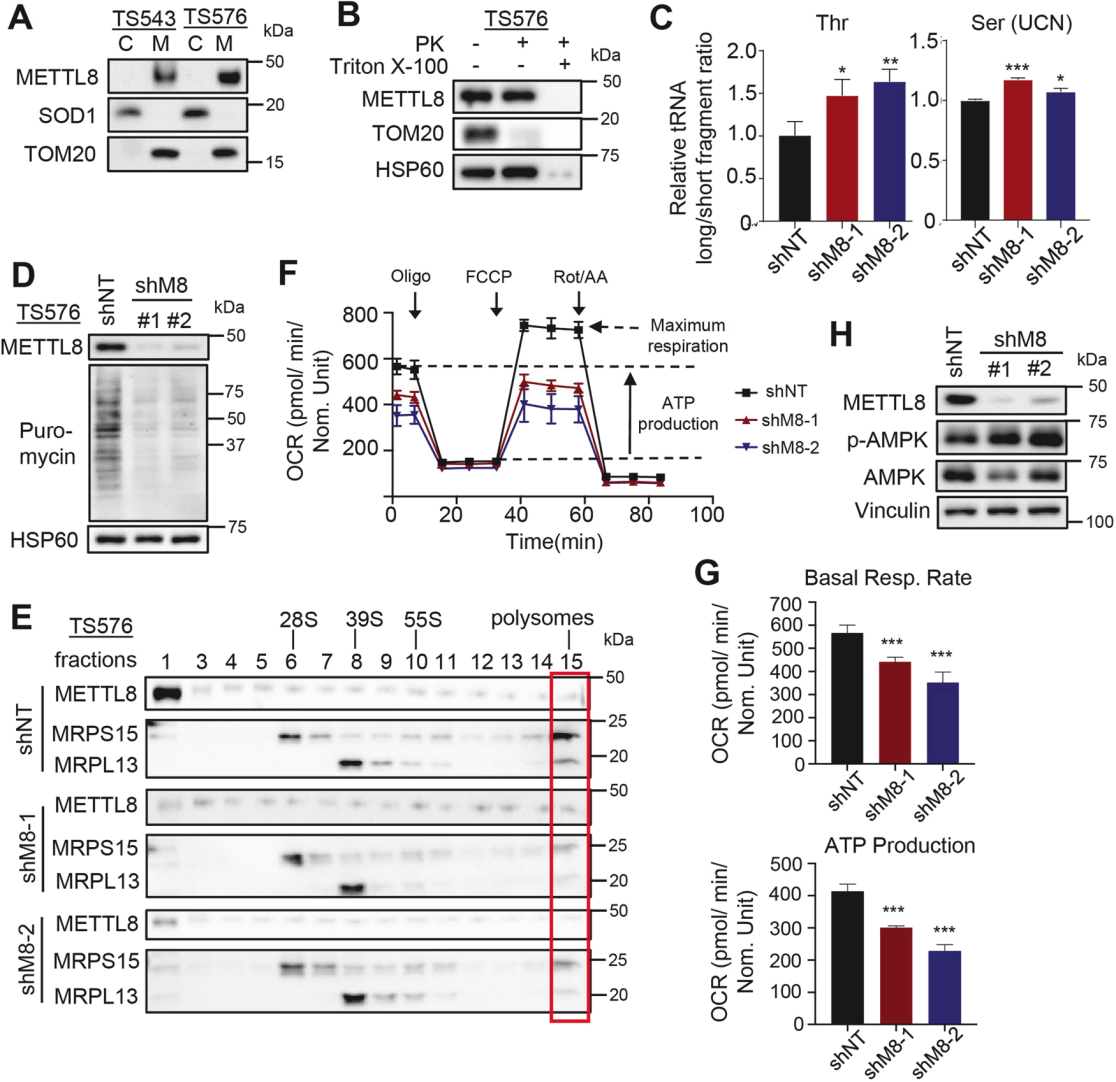
METTL8 mediates mt-tRNA m^3^C modification for mitochondrial translation and respiration in GSC. **A** Western blot analysis of METTL8 levels in the cytosolic (**C**) and mitochondrial (M) fractions of GSCs. **B** Western blot analysis of METTL8 levels upon proteinase K treatment of GSC mitochondrial extracts, with or without detergent. **C** qRT-PCR analysis of m^3^C modification on mt-tRNA^(Thr/Ser(UCN))^ upon *METTL8* KD (*n* = 3) (mean ± SD). *P < 0.05, **P < 0.01, ***P < 0.001. **D** Western blot analysis of puromycin levels in the mitochondrial extracts of *METTL8* KD GSC. **E** Western blot analysis of METTL8, MRPS15, and MRPL13 levels in different fractions of mitochondrial extracts of *METTL8* KD GSC after sucrose gradient ultracentrifugation. **F**, **G** Seahorse analysis of GSC upon *METTL8* KD (*n* = 3). ***P < 0.001. **H** Western blot analysis of p-AMPK and AMPK levels in *METTL8* KD GSC.

Next, we sought to address the subcellular localization of METTL8 in GSC. Using subcellular fractionation experiments, we demonstrate the exclusive mitochondrial localization of METTL8 in GSCs by performing western blot analysis with a polyclonal antibody against full length METTL8 that we have raised (Fig. 3A). Furthermore, only proteinase K treatment of GSC mitochondrial extracts in the presence of detergent led to METTL8 degradation, indicating that METTL8 resides in the GSC mitochondrial matrix (Fig. 3B). Using a published qPCR assay to assess the relative m^3^C modification levels [29], we found a significant increase in the long (unmethylated) vs short (methylated) fragments of mt-tRNA^Thr^ and mt-tRNA^Ser(UCN)^ in *METTL8* silenced GSC, consistent with the known mitochondrial function of METTL8 (Fig. 3C). *METTL8* silenced GSC correspondingly displayed reduced mitochondrial translation, which can be reported by the ribosome-catalyzed incorporation of puromycin (a naturally occurring aminonucleoside antibiotic that inhibits protein synthesis) into the C-terminus of elongating nascent chains (Fig. 3D). Furthermore, there were less actively translating polysomes in *METTL8* KD GSC based on sucrose gradient co-sedimentation experiments (Fig. 3E). Interestingly, METTL8 immunoprecipitation experiments showed that METTL8 bound to proteins of the mitoribosomal small and large subunits in a RNA-dependent manner, suggesting that METTL8 may associate with polysomes during mRNA translation (Supplementary Fig. 3A–C). Consistent with defective mitochondrial translation, oxygen consumption rate measurement of *METTL8* KD GSC showed a significant decrease in basal respiration and ATP production, along with increased AMPK phosphorylation (Fig. 3F–H). Given the link between mitochondrial fission and OXPHOS in GSCs, we wonder if *METTL8* depletion may affect mitochondrial fission, which can be indirectly reported by levels of p-DRP1^S616^ [30]. DRP1, a dynamin-like protein, is a crucial mediator of mitochondrial fission. *METTL8* silenced GSCs show decreased levels of p-DRP1^S616^, but no change in the level of p-DRP1^S637^ (an inhibitory modification), suggesting reduced mitochondrial fission (Supplementary Fig. 3D). We also used a published pathway classification of GBM to understand if *METTL8* expression may be restricted to the mitochondrial GBM [31]. There was no significant difference in *METTL8* expression in mitochondrial GBM when compared to the proliferative/progenitor, neuronal or glycolytic/plurimetabolic GBM (Supplementary Fig. 3E). Taken together, our data indicate a pivotal role of METTL8 in mediating mt-tRNA m^3^c modification for mitochondrial translation and respiration in GSC.

### *METTL8* loss inactivates RTK signaling via HIF1α downregulation in GSC

**Fig. 4:**
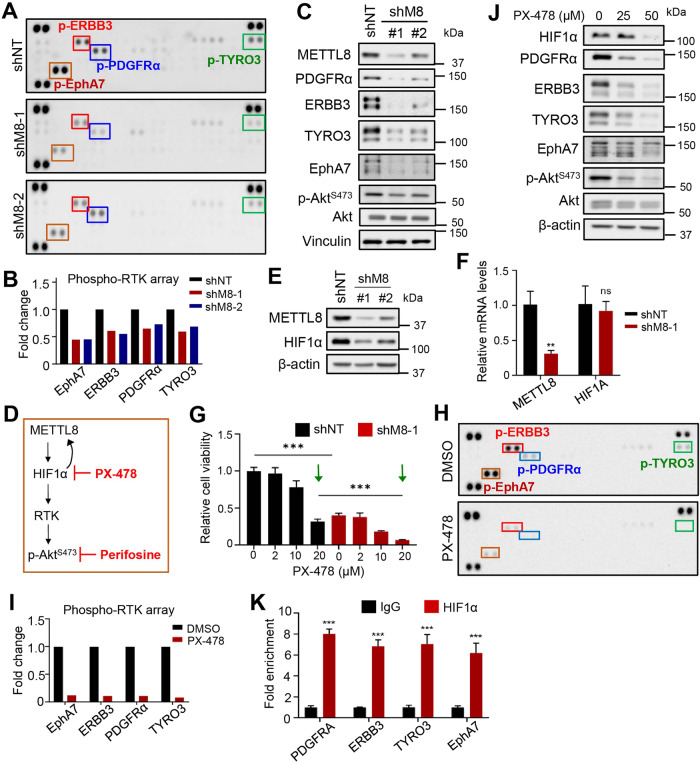
*METTL8* loss inactivates RTK signaling via HIF1α downregulation in GSC. Phospho-RTK array analysis of *METTL8* KD (**A**, **B)** or PX-478 treated (1 day) (**H**, **I**) GSC TS576. Representative blots (*n* = 2 replicates) (**A**, **H**) and quantification of EphA7, ERBB3, PDGFRα and TYRO2 dot intensities when normalized to the intensity of control dots (**B**, **I**) are shown. **C**, **J** Western blot analysis of PDGFRα, ERBB3, TYRO3, EphaA7, p-Akt^S473^, and Akt levels in *METTL8* KD (**C**) or PX-478 treated (**J**) GSC TS576. **D** Schematic diagram of the proposed mechanism. **E** Western blot analysis of HIF1α levels in *METTL8* KD GSC TS576. **F** qRT-PCR analysis of *HIF1A* mRNA levels in *METTL8* KD GSC. *HSP70* serves as the housekeeping genes (*n* = 3) (mean ± SD). **G** Cell viability of *METTL8* depleted GSC TS576, with or without PX-478 treatment (2 days). The values were normalized to the DMSO control (*n* = 6) (mean ± SD) ***P < 0.001. **K** ChIP-qPCR analysis of HIF1α enrichment at the promoters of *PDGFRA*, *ERBB3*, *TYRO3* and *EPHA7* in GSC TS576 (*n* = 3) (mean ± SD). ***P < 0.001 (*n* = 3) (mean ± SD).

Our data above indicated that METTL8 may be a good therapeutic target in GBM treatment but METTL8 inhibitors do not currently exist. Thus, we explored *METTL8* depletion-associated molecular alterations in GSC with the aim of identifying pharmacologically actionable nodes. Phospho-receptor tyrosine kinase (RTK) array analysis revealed decreased phosphorylation of multiple RTKs, including PDGFRα, ERBB3, TYRO3 and EphA7, in *METTL8* depleted GSC TS576 (Fig. 4A, B). The inactivation of these RTKs is associated with the downregulation of these RTKs (i.e. total protein levels) and was consistent with reduced Akt phosphorylation, which occurs downstream of RTK signaling (Fig. 4C). Since OXPHOS inhibitors reduce HIF pathway activity (due to increased intracellular oxygen via oxygen, prolyl hydroxylase- and VHL-dependent degradation of HIF1α [32–35]) and that HIF1α inhibition can downregulate PDGFRα signaling [36], we hypothesize that OXPHOS impairment upon *METTL8* silencing may downregulate HIF1α which in turn affects the expression of RTK genes (Fig. 4D). Indeed, *METTL8* depletion reduced HIF1α protein but not *HIF1A* mRNA levels in GSC TS576, which corroborated with the enhanced sensitivity of *METTL8* silenced GSC to PX-478 (Fig. 4E–G). Furthermore, PX-478 treatment (1 day to audit proximal changes) of GSC TS576 phenocopied *METTL8* depletion in reducing phosphorylation and total levels of PDGFRα, ERBB3, TYRO3 and EphA7 (Fig. 4H–J). The survey of publicly available HIF1α ChIP-Seq datasets revealed putative HIF1α binding sites at the promoters of *PDGFRA*, *ERBB3*, *TYRO3*, and *EPHA7*, which were validated in GSC TS576 using ChIP-qPCR assay (Supplementary Fig. 4A; Fig. 4K). Moreover, silencing each of these RTKs alone is sufficient to reduce Akt phosphorylation, suggesting that each of them can contribute to Akt signaling (Supplementary Fig. 4B). Correlative analysis of *METTL8* mRNA with cancer-related proteins in the TCGA RPPA (Reverse Phase Protein Array) glioma dataset unveiled significant positive correlations between *METTL8* levels and that of phospho- and total EGFR/ Akt, cementing the intimate link between METTL8 and the RTK/Akt pathway (Supplementary Fig. 4C). Collectively, our data support the view that *METTL8* loss-associated OXPHOS impairment in GSC reduces protein levels of HIF1α (likely by destabilizing HIF1α) that regulates RTK gene expression, thereby impairing the HIF1α/RTK/Akt axis.

### *METTL8* depleted GSCs are sensitive to Akt inhibitor, leading to the rational combination of HIF1α/Akt inhibitors to target GSC

**Fig. 5:**
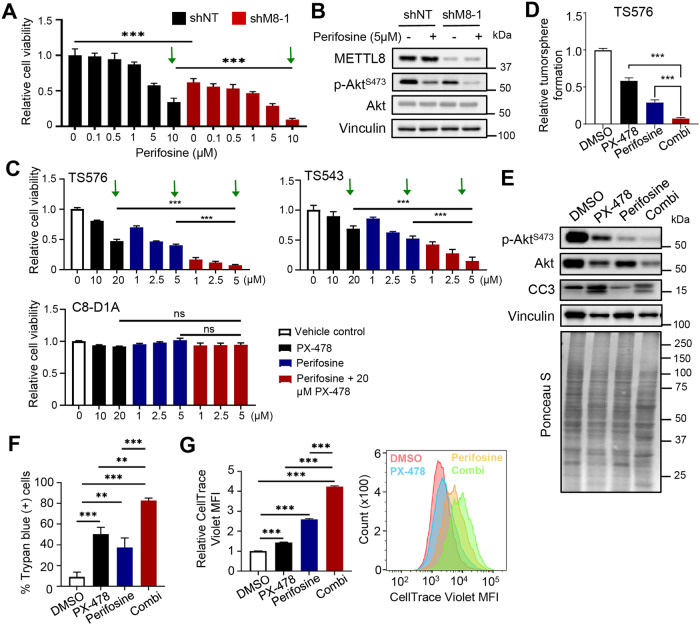
*METTL8* depleted GSCs are sensitive to Akt inhibitor, leading to the rational combination of HIF1α/Akt inhibitors to target GSC. **A** Cell viability analysis of shNT or shM8 transduced GSC, treated with or without Perifosine at the indicated concentrations for 2 days (*n* = 6) (mean ± SD). ***P < 0.001. **B** Western blot of p-Akt^S473^ and Akt levels in shNT or shM8 transduced GSC, with or without Perifosine treatment (5 µM, 2 days). **C** Cell viability assay of GSCs and mouse astrocytes with 3-day treatment of the indicated drugs (*n* = 6) (mean ± SD). ***P < 0.001. **D** Tumorsphere formation of GSC treated with the indicated drugs (5 days) (*n* = 6) (mean ± SD). ***P < 0.001. **E** Western blot analysis of p-Akt^S473^, Akt, cleaved-caspase 3 (CC3) levels with the respective drug treatment of GSC. **F** Trypan blue exclusion assay of GSC treated with the indicated drugs for 3 days. **P < 0.01, ***P < 0.001. **G** CellTrace^TM^ Violet staining of GSC treated with the indicated drugs (1 day) (*n* = 3) (mean ± SD). ***P < 0.001.

Since HIF1α inhibitor treatment phenocopies *METTL8* depletion in inactivating the RTK/Akt signaling in GSC, we explored the possibility that the co-inhibition of HIF1α and Akt may represent a new therapeutic approach against GSC. We first evaluated the response of GSC to Perifosine (an established Akt inhibitor that blocks Akt recruitment to the cell membrane [37]) in the presence or absence of METTL8 expression, and found that *METTL8* depleted GSC TS576 was more sensitive to Perifosine treatment than the *METTL8* intact counterpart (Fig. 5A, B). Next, we measured GSC viability upon PX-478, Perifosine or PX-478/Perifosine combination treatment after 3 days. Strikingly, the combination of 20 μM PX-478 and 5 μM Perifosine resulted in the greatest reduction in GSC cell viability when compared to single agent (combination: >85%; PX-478: 30–50%; Perifosine: 50–60%) (Fig. 5C). In contrast, there was no significant difference in cell viability of non-cancerous mouse astrocytes with the same dose of PX-478/Perifosine combination (Fig. 5C). Moreover, the PX-478/Perifosine combination synergistically reduced GSC tumorsphere formation, indicating loss of GSC self-renewal (Fig. 5D). This tracked with the greatest decrease in p-Akt^S473^ and Akt levels; frequency of dead cells (Trypan blue positive cells); and increase in the CellTrace™ Violet retention (~4-fold) in the drug combination treated GSC (Fig. 5E–G). Thus, the HIF1α/Akt inhibitor combination potently impedes proliferation/ self-renewal of GSC.

### The HIF1α/Akt inhibitor combination also suppresses proliferation of various hard-to-treat GBM cellular models in vitro

**Fig. 6:**
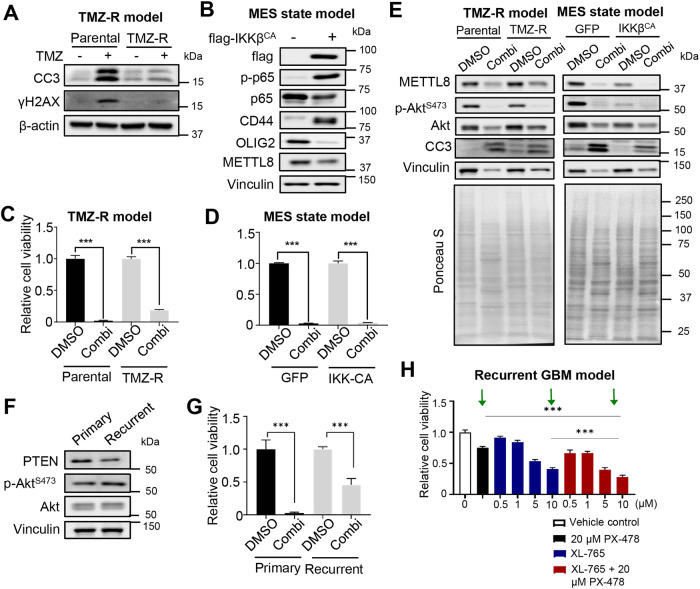
The HIF1α/Akt inhibitor combination also suppresses proliferation of various hard-to-treat GBM cellular models in vitro. **A** Western blot analysis of CC3 and γH2AX levels in parental vs TMZ-R GSCs, with or without TMZ treatment (200 µM, 5 days). **B** Western blot analysis of flag, p-p65, p65, CD44, OLIG2, and METTL8 levels in GSC overexpressing GFP or flag-IKKβ^CA^. **C**, **D** Cell viability assay of parental vs TMZ-R GSCs, or GFP vs flag-IKKβ^CA^ overexpressing GSCs, with or without PX-478/Perifosine combination treatment for 3 days (*n* = 6) (mean ± SD). ***P < 0.001. **E** Western blot analysis of METTL8, p-Akt^S473^, Akt, and CC3 in TMZ-R and mesenchymal GBM cells, with or without PX-478/Perifosine combination treatment for 3 days. **F** Western blot analysis of PTEN, p-Akt^S473^ and Akt levels in primary (G68-11) vs recurrent (G68-28) patient-derived GSC lines. **G** Cell viability assay of primary vs recurrent GSC lines, with or without PX-478/Perifosine combination treatment for 3 days (*n* = 6) (mean ± SD). ***P < 0.001. **H** Cell viability assay of recurrent GSC line (G68-28) when treated with the indicated drugs for 3 days (*n* = 6) (mean ± SD). ***P < 0.001.

To further evaluate the clinical utility of the PX-478/Perifosine combination, we assessed the efficacy of this drug combination in suppressing proliferation of several hard-to-treat GBM cellular models, including a Temozolomide-resistant (TMZ-R) GSC line, a mesenchymal GBM model for highly invasive and treatment-refractory GBM [26, 38], and a recurrent GBM patient-derived GSC line (National University Hospital, Singapore). In all cases, the drug combination effectively impaired GBM cell viability (Fig. 6A–D, F, G). We also confirmed the reduction in phospho-Akt levels for the TMZ-R GSC line and mesenchymal GBM model upon PX-478/Perifosine combination treatment (Fig. 6E). For the recurrent GBM line, a similar reduction in cell viability was observed with a combination of PX-478 and XL-765 (an established PI3K/mTOR dual inhibitor which targets Akt indirectly) (Fig. 6H). Collectively, we provide proof-of-concept that the PX-478/Perifosine combination may be further developed as a therapeutic option for GBM.

## Discussion

In this study, we provide compelling evidence of a reliance of GSCs on METTL8 to sustain their proliferation, stemness and tumorigenicity, which is clinically relevant. This differs from that reported in neural stem cells whereby *METTL8* absence favors cellular differentiation at the expense of self-renewal [29]. Although transcription factor YY1 regulates *METTL8* expression in breast cancer cells and STAT3 transcriptionally activates *METTL8* in mouse embryonic stem cells, the upstream regulator of METTL8 in GBM remains unknown [20, 21]. We demonstrate that *METTL8* overexpression in GSCs and GBM can be in part explained by H2AZ-mediated chromatin accessibility of HIF1α. This is substantiated by the presence of HIF1α ChIP-Seq peaks and a predicted HRE motif at the *METTL8* promoter; experimental validation of HIF1α enrichment at the *METTL8* promoter in GSC; and METTL8 downregulation upon pharmacological inhibition of HIF1α in GSC (Fig. 1D–I).

At the molecular level, we show that only mitochondrial METTL8 is detected in GSC, suggesting that *METTL8* isoform 1 is likely the most abundant isoform in GSC (Supplementary Fig. 5A). This exclusive subcellular localization of METTL8 corroborates with the reduction in mt-tRNA^Thr/Ser(UCN)^ m^3^C modification in *METTL8* silenced GSC, leading to decreased mitochondrial translation and respiration (Fig. 3). Although GSCs exhibit augmented OXPHOS activity (when compared to differentiated GSC) and rely on OXPHOS for energy production [39], our knowledge of the molecular mechanisms that underlie OXPHOS dysregulation in GSCs remains incomplete. An example is the RNA binding protein Imp2 that promotes OXPHOS activity in GSC by facilitating the delivery of mRNA encoding the subunits of electron transport chain to the mitochondria for translation [40]. Thus, METTL8-mediated mt-tRNA m^3^C modification represents another mechanism that enables OXPHOS upregulation in GSC.

Mechanistically, we describe a previously unrecognized HIF1α-METTL8 feedforward loop that sustains GSC stemness and tumorigenicity: HIF1α transcriptionally activates *METTL8*, and the METTL8-dependent OXPHOS in turn promotes HIF1α protein stability (Supplementary Fig. 5A). This aligns with previous reports showing a critical role of HIF1α in promoting GSC stemness and tumorigenicity [41], as well as IACS-010759 (an established OXPHOS inhibitor) treatment-induced HIF1α degradation that occurs in a prolyl hydrolase-dependent manner [42]. HIF1α downregulation upon *METTL8* loss subsequently weakens RTK/Akt signaling, which can be recapitulated by treating GSC with the HIF1α inhibitor PX-478 (Fig. 4). Notably, we show that *TYRO3* and *EPHA7*, in addition to *PDGFRA* and *ERBB3* (known HIF1α targets [43, 44]), are direct transcriptional targets of HIF1α in GSC, which can potentially account for the enigmatic RTK co-activation that occurs in GBM [45] (Supplementary Fig. 5A). Indeed, our identified RTKs have established roles in gliomagenesis. For instance, *PDGFRA* amplification occurs in ~13% GBM and PDGFB overexpression alone is sufficient to drive gliomagenesis [24, 46]); 9% of GBM tumors overexpress *ERBB3* via miR-205 inactivation [47]; and *EPHA7* overexpression portends poor patient prognosis in both primary and recurrent GBM [48]. Furthermore, Akt is one of the major downstream effectors of PDGFRα/ERBB3 signaling, and Akt inhibition reduces GSC self-renewal, invasiveness and tumorigenicity [49]. Given that dysregulated RTK signaling drives gliomagenesis in part by reprogramming cellular metabolism (e.g. glycolysis and cholesterol uptake) by engaging Akt-mTOR signaling [50], our findings underscore the complex crosstalk between metabolism and RTK signaling in GBM pathogenesis.

Since METTL8 indirectly controls mitochondrial translation and respiration in GSC, mitochondrial translation and OXPHOS inhibitors would conceptually mimic *METTL8* loss, but these compounds are unlikely suitable for GBM treatment due to the lack of knowledge as to whether they can pass through the blood-brain-barrier, their poor plasma stability, and toxicities [51–54]. In the absence of METTL8-specific inhibitors, our mechanistic investigation has led to the rational combination of HIF1α and Akt inhibitors to target GSC since METTL8 loss crippled the HIF1α/RTK/Akt axis (Supplementary Fig. 5A). In preclinical GBM models, PX-478 can overcome hypoxia-induced drug resistance to ferroptosis inducer, indicating that it can pass through the blood-brain-barrier [55]. Furthermore, a phase I clinical trial showed that PX-478 is well tolerated in patients with advanced solid cancers although its efficacy was not evaluated in GBM [56]. In a phase II clinical trial for recurrent GBM, Perifosine was found to be well-tolerated but exhibited limited efficacy as a monotherapy [37]. That the PX-478/Perifosine combination synergistically disabled GSC proliferation/ self-renewal in vitro and shows efficacy in several hard-to-treat GBM cellular models provide a strong rationale for future work to test this drug combination in pre-clinical GBM models (Figs. 5, 6). To improve drug delivery while minimizing toxicity, one can also consider loading the drug combination into nanocarriers (such as nanoparticles) for GBM treatment [57].

## Materials and methods

### Cell lines and compounds

Human GBM-derived GSCs (TS543, TS576, and TS603) were provided by Dr. Cameron Brennan (Memorial Sloan Kettering Cancer Center) and Dr. Ronald A. DePinho (MD Anderson Cancer Center) [58]. Human neural progenitor cells (hNPC) were induced from human embryonic stem cells H1 as described previously [59]. The GSCs and hNPC were cultured in human neural stem cell Maintenance Media (Millipore), 1% Penicillin-Streptomycin (PS), and supplemented with EGF and bFGF (20 ng/ml each), without B27. Non-cancerous mouse astrocytes (C8-D1A from ATCC) were provided by Dr. Thiruma V. Arumugam (La Trobe University) and cultured with DMEM/F12 with 10% FBS and 1% PS. U87 MG was kindly provided by Dr. Karen Crasta (National University of Singapore). U87 MG and HEK293T cells were cultured with DMEM-high glucose with 10% FBS and 1% PS.

Glioma tissue specimens were obtained with written informed consent as part of a study protocol approved by the National Healthcare Group Domain-Specific Review Board (NHG DSRB Ref: 2019/00068 and DSRB Ref: 2022/00103) and the National University of Singapore-Institutional Review Board (NUS-IRB-2022-22). Patient-derived gliomasphere (G68-08, G68-11, and G68-28) establishment and culture were performed as described previously [60, 61]. Briefly, freshly obtained glioma tissues were minced and incubated with enzymatic (Accutase™) dissociation solution. Dissociated cells contaminated with red blood cells (RBC) were lysed by short incubation with 1 x RBC lysis solution followed by subsequent washing before counting for live cells using trypan blue exclusion assay. Isolated live cells were culture in chemically defined serum-free medium supplemented with B27 (without vitamin A, Invitrogen), basic fibroblast growth factor (20 ng/ml, Peprotech), heparin (5 µg/ml, Sigma) and epidermal growth factor (20 ng/ml, Peprotech) in Dulbecco’s modified Eagle medium (DMEM)/ Ham’s F12 nutrient mix (F12, Gibco).

To establish Temozolomide-resistant GSC, early passage GSC TS576 was treated with 100 µM Temozolomide continuously for up to 2 months until the emergence of Temozolomide-resistant clones. Fresh media containing Temozolomide was replenished every 2 days. To generate the mesenchymal GBM model that is associated with enhanced invasiveness and treatment resistance, NF-kB signaling was activated in proneural GSC TS576 through FLAG-IKKβ^CA^ overexpression since NF-kB activation drives mesenchymal transformation in GBM [38, 62]. The mesenchymal transformation was confirmed by western blotting for protein levels of NF-kB p65 phosphorylation, CD44 (a well-established mesenchymal marker), and OLIG2 (a well-established proneural marker).

The following compounds were used in this study: PX-478 (MedChemExpress, HY-10231), Perifosine (MedChemExpress, HY-50909), HLM006474 (Selleckchem, S8963), S3I-201 (Selleckchem, S1155), Temozolomide (Sigma, T2577), and XL-765 (MedChemExpress, HY-15900), Nocodazole (Sigma, M1404), Forskolin (Sigma, F6886).

### DNA constructs

The shRNAs against human H2AZ2 (shH2AZ2#1, TRCN0000299143 and shH2AZ2#2, TRCN TRCN0000310400) and human METTL8 (shMETTL8#1, TRCN0000236515 and shMETTL8#2, TRCN0000236515) were purchased from Sigma. shRNAs against RTK genes were designed using the Broad Institute Genetic Perturbation Platform (GPP) portal (https://portals.broadinstitute.org/gpp/public/clone/search) and cloned into the pLKO.1 puro vector (Addgene, # 8453). plenti6.3-FLAG-IKKβ^CA^ construct was generated by subcloning the ORF from pCMV2-FLAG-IKKβ^CA^ (Addgene, #11105) into plenti6.3/V5-DEST vector. The non-targeting shRNA (shNT) was kindly provided by DePinho lab. Primers used for cloning are listed in Table S1.

### Lentiviral transduction

Lentiviruses were generated by co-transfecting HEK293T cells with pMD2.G, pRSV-Rev, pMD-VSVG and overexpression/shRNA plasmids. The media was collected 72-h post-transfection, concentrated using ultracentrifugation (Optima XL-100K) and the lentiviral particles were resuspended in DMEM/F12. GSCs were transduced with lentivirus in the presence of 0.4 µg/ml polybrene (Sigma) and the overexpression/knockdown efficiency was validated using Western blot analysis 72-h post transduction.

### In vitro limiting dilution and tumorsphere formation assays

GSCs were stained with propidium iodide (PI, Sigma), and PI-negative cells (*n* > 6) were flow-sorted with decreasing number of cells per well (1, 10, 25, and 100) plated in 96-well plates. The percentage of wells with tumorspheres was quantified after 7 days under a microscope. Extreme limiting dilution analysis was performed using software available at http://bioinf.wehi.edu.au/software/elda/. The tumorsphere formation assay involved seeding GSCs at a density of 1 cell per µl, and the number of tumorspheres in each well was quantified after 7 days. For drug treatment, GSCs were incubated with the respective inhibitors for 5 days, followed by tumorspheres scoring. Data presented are from six replicates.

### Anchorage-independent growth assay

Anchorage-independent growth assays were performed in replicates of four in six-well plates. Indicated cells were seeded (1 × 10^4^ cells per well) in stem cell proliferation media with EGF and βFGF containing 0.5% low-melting agarose on the top of bottom agar containing 1% low-melting agarose stem cell proliferation media with EGF and bFGF. After 14–21 days, colonies were stained with iodonitrotetrazoliumchloride (Sigma) and counted. Data presented are from four replicates.

### Transwell migration and invasion assay

The invasiveness of GSCs was measured using 6.5 mm Transwell with 8.0 µm pore polycarbonate membrane insert (Corning, CLS3422). The membrane was coated with Matrigel Basement Membrane Matrix (100 µg/cm^2^) (BD Biosciences). The cells were seeded in the upper compartment with serum-free GSC medium. The wells of the lower chamber were filled with GSC medium containing 10% FBS. At the end of the invasion assay, chambers were removed, fixed, and stained with a 0.5% Crystal Violet. Cells on the upper surface of the filters were removed by wiping with a cotton swab, and invasion was determined by counting the cells that migrated to the bottom side of the filter using at least ten fields per insert at ×20 magnification. Each assay was performed in triplicate.

### In vitro EdU (5-ethynyl-2’-deoxyuridine) labeling

Transduced GSCs were seeded onto coverslips and incubated with 10 µM EdU (Toronto Research Chemicals) for 1 h at 37 °C. The cells were fixed with 4% paraformaldehyde, followed by blocking with immunofluorescence blocking buffer (10% FBS, 1% BSA, 0.3% Triton-X100). After that, the cells were stained with EdU staining solution (100 mM Tris pH 7.5, 4 mM CuSO_4_, 1 mg/ml Sulfo-Cyanide Azide, 100 mM Sodium Ascorbate) for 1 h at room temperature. Images were captured with a Leica DCF 9000 GT digital camera, using a Leica DMi8 microscope. The data presented are from two independent experiments with similar results.

### Seahorse assay

Cells were plated at optimal densities in Seahorse XF 24-well plates one or two days prior to the measurement. Cells were incubated with Seahorse XF Assay Media at 37 °C for 1 h without CO_2_ for basal OCR and with MAS buffer (Mannitol and sucrose buffer: 70 mM sucrose, 220 mM Mannitol, 10 mM KH2PO4, 5 mM MgCl2, 2 mM HEPES, and 1 mM EGTA in diH2O. pH 7.2 using 0.1 M KOH) to measure complex activity just before running the assay. Substrate concentrations were 1μM for Oligo and FCCP, 1μM/0.5μM for Rot/AA, and 5 mM for succinate, all the substrates were purchased from Seahorse Bioscience. Reagents for complex activity such as Saponin 100 μg/ml, Pyruvate 10 mM, Malate 2 mM, ADP 50 μM and NADH 10 mM were purchased from Sigma. OCR measurements were obtained using the Seahorse XFe24 Analyzer, and normalized to protein concentration (µg/µL).

### RNA isolation and RT-qPCR

Detection of m^3^C modification of mt-tRNAs was performed as previously described [29]. Briefly, RNA was extracted using RNeasy^®^ Mini or Micro Kit (Qiagen) and then cDNA synthesis was performed with each mt-tRNA^Thr^ and mt-tRNA^Ser(UCN)^ specific primer. To examine *HIF1A* expression upon METTL8 silencing, cDNA synthesis was performed using Superscript^TM^ III First-Strand Synthesis System (Invitrogen). RT-qPCR was performed using PowerUp™ SYBR® Green Master Mix (Applied Biosystems). Each assay was performed in triplicate. *HSP70* was used as housekeeping gene. Primers used for qPCR are listed in Table S2.

### Western blot analysis and antibodies

Whole cell lysates were prepared in RIPA buffer (Thermo) with protease inhibitor (Roche), and phosphatase inhibitor (Roche). Protein concentration was determined by DC Protein Assay (Bio-rad), and equal amount of protein samples was used to perform SDS gel electrophoresis and transferred onto nictrocellulose membranes (Bio-rad). TBST with 5% skim milk was used for blocking. Incubation with primary antibody was performed at 4 °C for 16 h. For the loading control of drug treatment experiments, nitrocelluloase membranes were stained with the Ponceau S solution (Sigma) after wet transfer. The antibodies are listed in Table S3.

### Immunoprecipitation

For IP, cell lysates were resuspended in an appropriate volume of IP lysis buffer (50 mM Tris, pH 7.4, 300 mM NaCl, 1% Triton X-100, protease inhibitor, phosphatase inhibitor) and subjected to 10 cycles (10 seconds on, 30 s off) of sonication (Bioruptor Plus, Diagenode). Samples were then centrifuged at 13,000 rpm for 15 min and the supernatant was collected. To pre-clear the lysate, 10 mg of supernatant was rotated with 25 μl protein A/G PLUS Beads (Santa Cruz) and 10 μg of anti-myc antibody (Cell Signaling Technologies) overnight at 4 °C. The next day, the agarose beads were washed thrice with IP wash buffer (50 mM Tris, pH 7.4, 300 mM NaCl, 1% Triton X-100). Finally, the beads were incubated in 25 μl of 2× protein dye at 95 °C for 10 min to dissociate bound proteins or the bound beads were sent for mass spectrometry analysis. Equal volumes of samples were loaded onto 15% SDS-PAGE gel and proteins were separated by molecular weight by gel electrophoresis. After which, Western blotting was performed.

### ChIP-qPCR analyses

Briefly, cells were cross-linked with 1% formaldehyde for 10 min at room temperature. The cells were lysed using SDS Lysis buffer for ChIP (1% SDS, 10 mM EDTA, 50 mM Tris-HCl pH 8). The lysate was sonicated and diluted in ChIP dilution buffer (0.01% SDS, 1% Triton X-100, 1.2 mM EDTA, 16.7 mM Tris-HCl pH 8, 167 mM NaCl) and used for the immunoprecipitation with rabbit IgG, anti-H2AZ (Abcam, 9139) and anti-HIF1α (Novus Biologicals, NB100-479) antibodies for ChIP-qPCR with protein A/G agarose beads (Pierce) or Dynabeads™ Protein G (Thermo). After an overnight incubation with antibody, the bound DNA was washed sequentially with low salt wash buffer (0.1% SDS, 1% Triton X-100, 2 mM EDTA, 20 mM Tris-HCl pH 8, 150 mM NaCl), high salt wash buffer (0.1% SDS, 1% Triton X-100, 2 mM EDTA, 20 mM Tris-HCl pH 8, 500 mM NaCl), LiCl wash buffer (0.25 M LiCl, 1% NP40, 1%deoxycholate, 1 mM EDTA, 10 mM Tris-HCl pH 8) and TE wash buffer (10 mM Tris-HCl pH 8, 1 mM EDTA) to remove non-specific sequences and eluted in the elution buffer (84 mg NaHCO3, 1 ml 10% SDS, 9 ml H2O). Then the samples were reverse cross-linked using NaCl at 65 °C overnight. The eluted DNA was purified and ChIP-qPCR was performed using PowerUp™ SYBR® Green Master Mix (Applied Biosystems). ChIP-qPCR primer sequences are listed in Table S4.

### Cell viability assay

Briefly, GSCs or mouse astrocytes were seeded on the 96 well plates, followed by drug treatment for 72 h. The Cell viability was measured using the CellTitre-Glo (Promega) assay according to the protocols specified by the manufacturer. Data was normalized to DMSO control. The data presented are from six replicates.

### Intracranial tumor formation in vivo

GSCs (7.5 × 10^5^ viable cells) were grafted intracranially into NSG mice (InVivos) aged 6–8 weeks. Tumor incidence was determined at indicated timepoints by luciferase imaging of mice using Xenogen IVIS (PerkinElmer) according to manufacturer’s instructions. Animals were maintained until neurological signs were apparent, at which point they were sacrificed. All animal procedures were performed in accordance to a protocol approved by the National University of Singapore Institutional Animal Care and Use Committee.

### Mitochondria fractionation

Whole cells were harvested, washed with 1 × PBS and resuspended with mitochondria buffer A (200 mM Mannitol, 68 mM Sucrose, 50 mM Pipes-KOH pH 7.4, 50 mM KCL, 5 mM EGTA, 2 mM Mgcl_2_, 1 mM DTT, Protease inhibitor and Phosphatase inhibitor). Resuspension was incubated on ice for 20 min. A Dounce Homogeniser was used to lyse the cells and samples were subsequently centrifuged at 300 × *g* for 10 min at 4 °C. The supernatant was taken and further centrifuged at 10,000 × *g* for 10 min at 4 °C. The resulting supernatant was taken as the cytosolic fraction and the pellet as the whole mitochondria.

For protein K treatment, whole mitochondria pellet was treated with 2 µg/ml of proteinase K only or 2 µg/ml of proteinase K and 1% Triton X-100 (Sigma) for 20 min on ice. 2 mM of PMSF was added to stop the reaction. Samples were spun down and resuspended in buffer A for quantification and Western blot analysis.

To assess mitochondrial translation, whole mitochondria pellet was resuspended in NSC medium containing 10 µg/µl of puromycin (Sigma) for pulse labelled for 10 min at 37 °C. Samples were spun down, resuspended in fresh medium and incubated for 30 min at 37 °C. After incubation, samples were pelleted again and resuspended in buffer A for quantification and Western blot analysis.

### Sucrose gradient ultracentrifugation

For mitoribosome isolation, 2 mg of mitochondria were used for each gradient. Mitochondria were defrosted on ice and lysed in two volumes of lysis buffer (25 mM HEPES–KOH, pH 7.5, 100 mM KCl, 20 mM MgCl2, 1% Triton X-100, 1 mM DTT, protease inhibitor, phosphatase inhibitor) for 10 min on ice. Lysates were cleared by centrifugation at 16,000 × *g* for 30 min at 4 °C and subsequently loaded on a 10 ml of 10–30% sucrose gradient (25 mM HEPES–KOH, pH 7.5, 100 mM KCl, 20 mM MgCl2, 1% Triton X-100, 1 mM DTT, protease inhibitor, phosphatase inhibitor) and centrifuged in SW41Ti rotor at 24,000 rpm for 16 h. Mitoribosome gradients were fractionated into 15 fractions using a piston gradient fractionator (BioComp) with monitoring absorbance at 260 nm. Protein samples from each fraction were analysed by western blot.

### Phospho-RTK array analysis

RTK phosphorylation was quantified using a Human Phospho-RTK Array Kit (R&D Systems, ARY001B). Briefly, GSC TS576 were transduced with non-targeting and METTL8 shRNAs or treated with 50 µM PX-478 for 24 h. GSCs were lysed in lysis buffer and 4–5 mg of protein was incubated overnight with the phospho-kinase array membrane at 4 °C, and then the membrane was incubated with an HRP-conjugated anti-phosphotyrosine detection antibody. Quantification of protein expression was performed using Image J.

### CellTrace^TM^ violet staining

GSCs were treated with the respective inhibitors for 24 h. Then, the cells were harvested and labeled with 5 µM Cell Trace^TM^ Violet dye (Invitrogen) according to the protocols specified by the manufacturer. The labelled GSCs were incubated for 7 days, followed by flow cytometry analysis using the Analyser Fortessa. The mean fluorescence intensity (MFI) was calculated using FlowJo software and data presented was from three replicates.

### Public datasets and data analyses

Processed tumor gene expression and clinical data for TCGA (https://www.cancer.gov/tcga), REMBRANDT cohorts have been obtained from GlioVis portal (http://recur.bioinfo.cnio.es). Gene expression and clinical data for Chinese Glioma Genome Atlas (CGGA) glioma patient cohort was downloaded from (http://www.cgga.org.cn/ download.jsp, DataSet ID:mRNAseq_693).

Public TS543 GSC ATAC-seq and ChIP-seq data for H2AZ, H3K4me3, H3K27ac used in the study have been accessed from GEO: GSE152858 and GSE152862 [7, 8]. HIF1α ChIP-Seq public processed data have been obtained from GSM2257670 (U2OS cells) and from GSM2835770 (PC3 cells). Raw public data for 4 random GBM patient ChIP-seq samples (H3K27Ac) have been accessed from GSE119755 [63]. Raw ChIP-sequencing data from 4 different regions of normal brain specimens were accessed through the ENCODE and Roadmap Epigenomics projects [64] and reanalysed. The data have been processed as follows. Reads were filtered based on quality and adapter sequences were removed from the ChIP-seq experiments using Trim_galore (https://github.com/FelixKrueger/TrimGalore) with the default options. The resulting trimmed fastq files were aligned to the human reference genome (hg19) using STAR_2.5.0a (https://github.com/alexdobin/STAR) with the following parameters: “--alignIntronMax 1”, “--outFilterMismatchNoverLmax 0.09”, “--alignMatesGapMax 2000”, “--outFilterMultimapNmax1”, “--alignEndsType EndToEnd“; the rest of the options were set to the default. Duplicated reads were removed from the bam files using MarkDuplicates software(http://broadinstitute.github.io/picard/). Newly generated BAM files have been processed into RPKM normalized BigWig files and visualized using Integrative Genomic Viewer (https://software.broadinstitute.org/software/igv/).

Putative hypoxia-response elements prediction at the *METTL8* proximal promoter (−3000/+50 bp from TSS) was done using consensus motif sequence (5′-A/GCGTG-3′) obtained from Chen et al. [65] as an input using FIMO tool from MEME suite (https://meme-suite.org/meme/tools/fimo).

Upgraded TCGA GBM patients classification information into glycolytic/plurimetabolic (GPM), mitochondrial (MTC), neuronal (NEU) and proliferative/progenitor (PPR) subtypes (Fig. S3D) was obtained from [31].

TCPA portal (v3.0) was used to download TCGA glioma patients RPPA level 4 data https://tcpaportal.org/tcpa/download.html for EGFR, EGFR_pY1068, EGFR_pY1173, AKT, AKT_pS473 and AKT_pT308 protein expression. The protein expression data was compared with *METTL8* mRNA expression data available for the same patients from Gliovis portal (http://gliovis.bioinfo.cnio.es [66]). The low expression and the high expression glioma patients subgroups have been generated as the bottom 25% lowest protein expressors and the top 25% highest protein expressers in TCGA glioma tumors. R package ggpubr was used for generation of correlation plots.

One-dimensional data-driven grouping (1‐D DDg) method was used to estimate whether the expression of gene of interest was significantly associated with cancer patient’s survival [67, 68]. After sorting the patient data by the gene expression values, the values were fitted to survival times and events using the Cox proportional hazards model; goodness-of-fit analysis was applied to get the separation between the sorted patients into low- and high-risk subgroups. The Cox hazards model and Wald test statistic were used to compute the differences between the Kaplan-Meier survival curves. Survival curves were visualized using R package survminer.

### Proteomics sample preparation

METTL8 samples were prepared for proteomics analysis by IP with mitochondria fraction. Samples were washed with phosphate-buffered saline (PBS) twice and supernatant was removed completely. Beads were then resuspended in 50% (v/v) trifluoroethanol (TFE) in 50 mM triethylammonium bicarbonate (TEAB), pH 8.5 containing 10 mM final concentration of tris(2-carboxyethyl)phosphine (TCEP) and incubated for 20 min at 55 °C for disulfide bridge reduction. Samples were cooled to 25 °C and alkylated with 55 mM 2-chloroacetamide (CAA) in the dark for 30 min, followed by on-bead digestion with endoproteinase LysC (2 µg final amount) for 3 h and subsequently by trypsin (2 µg final amount) at 37 °C overnight. Once completed, beads were removed and the peptides were transferred to new tubes. Digestion was terminated by adding 1% (v/v) final concentration of trifluoroacetic acid (TFA) to the samples, followed by desalting using C18 StageTips. Desalted peptides were dried by centrifugal evaporation, resuspended in 25 µl of TEAB, pH 8.5, and individually labelled using isobaric 6-plex tandem mass tags (TMT6-plex, Thermo Fisher Scientific) at 25 °C overnight. TMT-126, 128, 130, and 131 tags were used. After labelling was completed, the reaction was quenched by addition of 30 µl of 1 M ammonium formate, pH 10 into each tube before pooling the samples into a new low-binding 1.5-ml microfuge tube. Pooled sample was desalted and fractionated on a self-packed spin column containing C18 beads (Dr Maisch GmbH) using 18%, 26%, and 60% acetonitrile in 10 mM ammonium formate, pH 10 as the step gradients. Fractions were dried by centrifugal evaporation and further washed and dried twice by addition of 60% acetonitrile in 0.1% formic acid to further remove residual ammonium formate salts.

### Protein interactomics by tandem mass spectrometry analysis

Dried fractions were resuspended in 30 µl of 2% (v/v) acetonitrile containing 0.06% (v/v) trifluoroacetic acid and 0.5% (v/v) acetic acid and transferred to an autosampler plate. Online chromatography was performed in an EASY-nLC 1000 (Thermo Fisher Scientific) liquid chromatography system using a single-column setup and 0.1% formic acid in water and 0.1% formic acid in 99% acetonitrile as mobile phases. Fractions were injected and separated on a reversed-phase C18 analytical column (Easy-Spray, 75 µm inner diameter × 50 cm length, 2 µm particle size, Thermo Fisher Scientific) maintained at 50 °C and using a 2–25% (v/v) acetonitrile gradient over 53 min, followed by an increase to 60% over the next 10 min, and to 90% over 2 min. The final mixture was maintained on the column for 5 min to elute all remaining peptides. Total run duration for each sample was 70 min at a constant flow rate of 300 nl/min.

Data were acquired using an Orbitrap Fusion mass spectrometer (Thermo Fisher Scientific) using data-dependent mode. Samples were ionized using 2.1 kV and 300 °C at the nanospray source. Positively-charged precursor signals (MS1) were detected using an Orbitrap analyzer set to 60,000 resolution, automatic gain control (AGC) target of 400,000 ions, and maximum injection time (IT) of 100 ms. Precursors with charges 2–7 and having the highest ion counts in each MS1 scan were further fragmented using higher-energy collision dissociation (HCD) at 42% normalized collision energy. Fragment signals (MS2) were analysed by the Orbitrap analyzer at a resolution of 7500, AGC of 80,000 and maximum IT of 22 ms. Precursors used for MS2 scans were excluded for 60 s to avoid re-sampling of high abundance peptides. The MS1–MS2 cycles were repeated every 3 s until completion of the run.

### Proteomics data analysis

Peak list was generated by Proteome Discoverer™ (v2.3, Thermo Fisher Scientific) and proteins were identified using Mascot search engine (v2.6.1, Matrix Science Ltd). Raw mass spectra were searched against human primary protein sequences retrieved from Swiss-Prot (11 June 2019). Carbamidomethylation on Cys and TMT6-plex on Lys and N-terminus were set as a fixed modification; deamidation of asparagine and glutamine, acetylation on protein N-termini, and methionine oxidation were set as dynamic modifications for the search. Trypsin/P was set as the digestion enzyme and was allowed up to three missed cleavage sites. Precursors and fragments were accepted if they had a mass error within 20 ppm and 0.06 Da, respectively. Peptides were matched to spectra at a false discovery rate (FDR) of 1% (strict) and 5% (relaxed) against the decoy database and quantitated using TMT6-plex method. Search result was exported and further processed for differential analysis using an in-house R-based script that was built upon the limma package (Ritchie et al., 2015) from Bioconductor. Proteins with differential expression were identified by comparing the treatment with the control with a log2 fold change (log2 FC) cutoffs of 1 and –1 and *p* value adjusted using the Benjamini-Hochberg method of <0.05 as significant hits.

NOTES Mass spectrometry proteomics data have been deposited to the Japan ProteOme STandard Repository (jPOSTrepo) with the dataset identifier (JPST002087).

### Statistical analyses

All the quantitative data were presented as mean ± standard deviations as described in the figure legends. For computing the statistical significance, Student *t*-test and One-way ANOVA were performed using Graph Pad Prism (Version 9.3.1) or Wilcoxon-Man–Whitney test using Cytel studio (Version 9.0.0). Significance was defined as *P* < 0.05.

### Supplementary information

Supplementary Material

Supplementary Table

Figure S1

Figure S2

Figure S3

Figure S4

Figure S5

Source data for Figures

Source data for Supplementary Figures

## Data Availability

All data, supplemental data, and data in repositories are available. Raw data from METTL8 immunoprecipitation-mass spectrometry analysis is available on Japan ProteOme STandard Repository (jPOSTrepo) with the dataset identifier (JPST002087). All other data and materials are available from the corresponding author upon request.
