# Author Correction: METTL8 links mt-tRNA m^3^C modification to the HIF1α/RTK/Akt axis to sustain GBM stemness and tumorigenicity

**DOI:** 10.1038/s41419-024-06917-x

**Published:** 2024-07-31

**Authors:** Bernice Woon Li Lee, You Heng Chuah, Jeehyun Yoon, Oleg V. Grinchuk, Yajing Liang, Jayshree L. Hirpara, Yating Shen, Loo Chien Wang, Yan Ting Lim, Tianyun Zhao, Radoslaw M. Sobota, Tseng Tsai Yeo, Andrea Li Ann Wong, Kejia Teo, Vincent Diong Weng Nga, Bryce Wei Quan Tan, Toshio Suda, Tan Boon Toh, Shazib Pervaiz, Zhewang Lin, Derrick Sek Tong Ong

**Affiliations:** 1 Department of Physiology, Yong Loo Lin School of Medicine, National University of Singapore, Singapore, 117593 Singapore; 2 NUS Center for Cancer Research, Yong Loo Lin School of Medicine, National University of Singapore, Singapore, Singapore; 3 Cancer Science Institute of Singapore, National University of Singapore, Singapore, 117599 Singapore; 4 The N.1 Institute for Health, National University of Singapore, Singapore, Singapore; 5 The Institute for Digital Medicine (WisDM), Yong Loo Lin School of Medicine, National University of Singapore, Singapore, Singapore; 6 Functional Proteomics Laboratory, SingMass National Laboratory, Institute of Molecular and Cell Biology, Agency for Science, Technology and Research (A*STAR), Singapore, Singapore; 7 Department of Surgery, Division of Neurosurgery, National University Hospital, Singapore, Singapore; 8 Department of Haematology-Oncology, National University Hospital, Singapore, Singapore; 9 Department of Medicine, National University Hospital, Singapore, Singapore; 10 International Research Center for Medical Sciences, Kumamoto University, Kumamoto, 860-0811 Japan; 11 Healthy Longevity Translational Research Programme, Yong Loo Lin School of Medicine, National University of Singapore, Singapore, Singapore; 12 Department of Biological Sciences, 14 Science Drive 4, National University of Singapore, 117543 Singapore, Singapore; 13 Institute of Molecular and Cell Biology (IMCB), Agency for Science, Technology and Research (A*STAR), Singapore, Singapore; 14 National Neuroscience Institute, 308433 Singapore, Singapore

**Keywords:** CNS cancer, RNA modification

Correction to: *Cell Death and Disease* 10.1038/s41419-024-06718-2, published online 14 May 2024

In this article, Figs. 1 and 2 have been corrected.

Figure 1C: the legends are missing. This has been corrected.

Figure 1B: the colors of the bars are missing. This has been corrected.

Corrected Figure 1
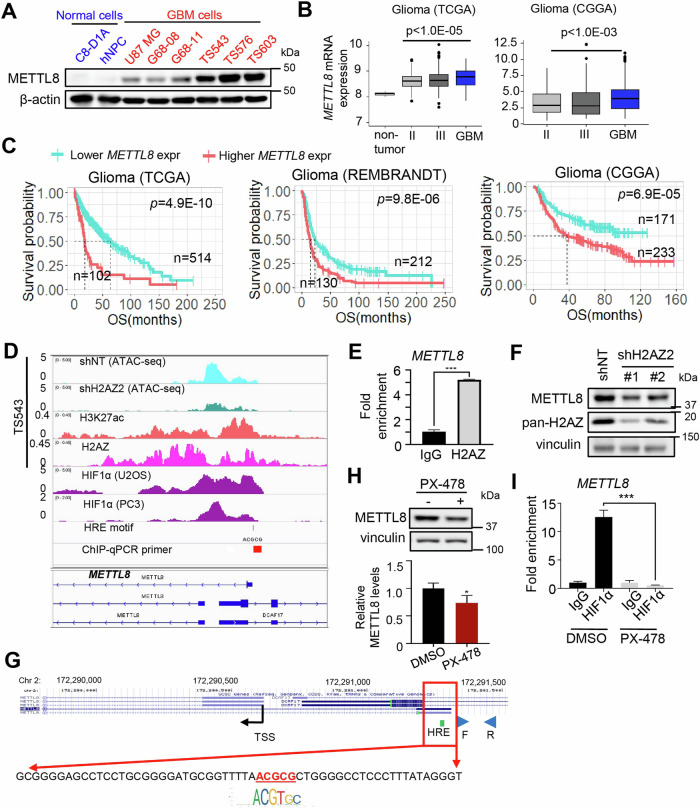


Original Figure 1
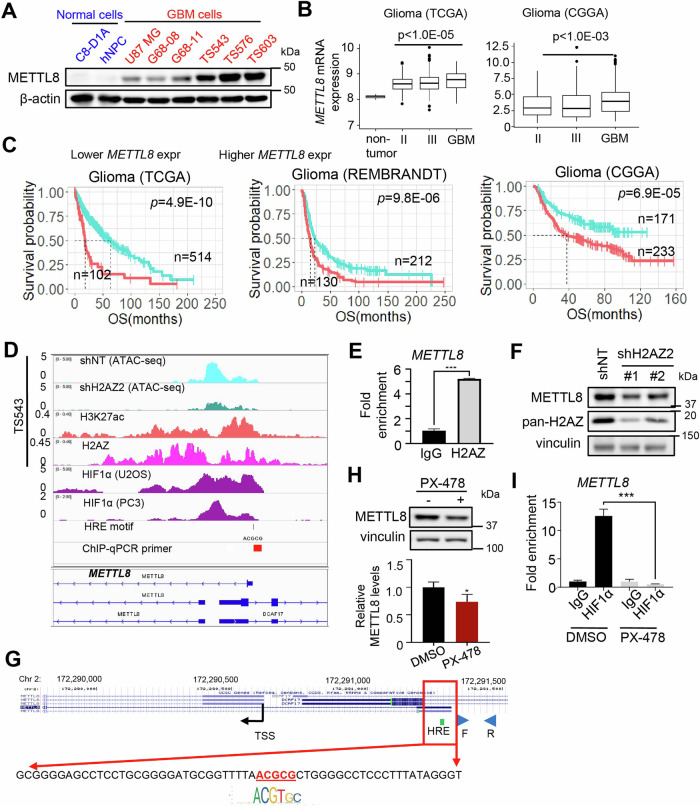


Figure 2B: the red lines for shM8-1 are missing. This has been corrected.

Corrected Figure 2
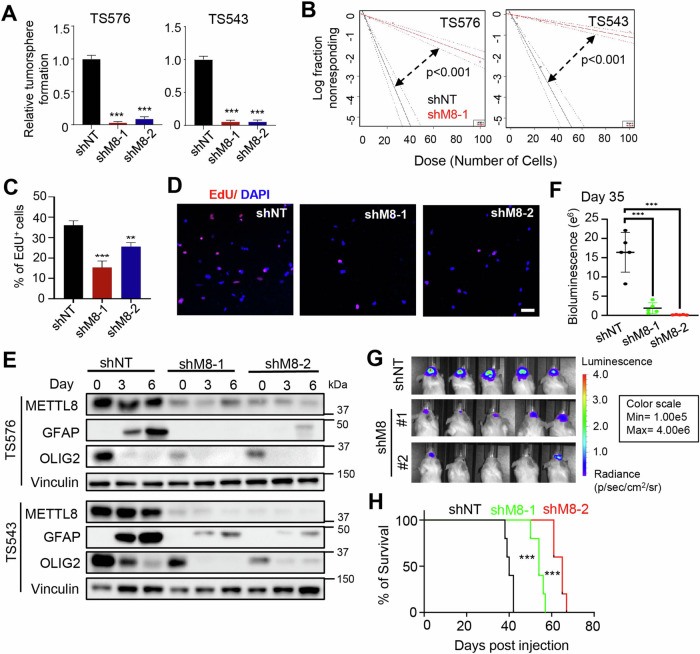


Original Figure 2
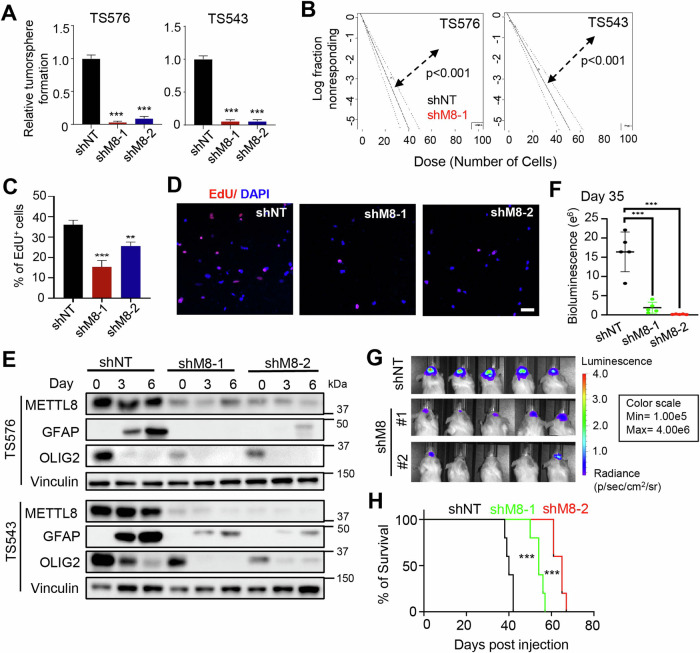


The original article has been corrected.

