# Supplementary material for: METTL8 links mt-tRNA m3C modification to the HIF1α/RTK/Akt axis to sustain GBM stemness and tumorigenicity
Source: Cell Death Dis. 2024 May 14;15(5):338. doi: 10.1038/s41419-024-06718-2 (PMC11093979; doi:10.1038/s41419-024-06718-2)
Supplement: Supplementary file 1 — Supplementary Material [file 41419_2024_6718_MOESM1_ESM.docx]

**SUPPLEMENTARY INFORMATION**

**METTL8 links mt-tRNA m^3^c modification to the HIF1α/RTK/Akt axis to sustain GBM stemness and tumorigenicity**

Bernice Woon Li Lee^1,2†^, You Heng Chuah^1,2†^, Jeehyun Yoon^1,2†^, Oleg V. Grinchuk^1,2†^, Yajing Liang^1^, Jayshree L Hirpara^3^, Yating Shen^4,5^, Loo Chien Wang^6^, Yan Ting Lim^6^, Tianyun Zhao^6^, Radoslaw M Sobota^6^, Tseng Tsai Yeo^7^, Andrea Li Ann Wong^8^, Kejia Teo^7^, Vincent Diong Weng Nga^7^, Bryce Wei Quan Tan^9^, Toshio Suda^3,10^, Tan Boon Toh^4,5^, Shazib Pervaiz^1,2,11^, Zhewang Lin^12^, Derrick Sek Tong Ong^1,2,13,14^*

**Supplementary figure legends**

**Supplementary Fig. S1. GSCs overexpress *METTL8* via H2AZ-mediated chromatin accessibility of HIF1α.** **(A)** Visualization of *METTL8* expression in different cell types derived from GBM tumors using GBMseq (<http://gbmseq.org/search>). **(B)** H3K27ac ChIP-Seq tracks at the *METTL8* promoter in GBM vs normal brain tissues. **(C)** Correlative analysis of *METTL8* mRNA levels with that of GBM-relevant transcription factors in multiple glioma patient cohorts (Gliovis). **(D)** Western blot analysis of METTL8 levels in GSC treated with or without HLM006474 (15µM, 1 day). PLK1 serves as the positive control while vinculin serves as the loading control. The relative METTL8 levels was normalized to DMSO control (n=3) (mean ± SD). **(E)** Western blot analysis of METTL8 levels in GSC treated with or without S3I-201 (75µM, 3 days). p-STAT3^Y705^ serves as positive control whereas vinculin serves as loading control. The relative METTL8 levels was normalized to DMSO control (n=4) (mean ± SD).

**Supplementary Fig. S2. *METTL8* depletion impairs GSC proliferation, transforming potential and invasiveness. (A)** Western blot analysis of CC3 levels in GSCs upon *METTL8* KD. METTL8 serves as the positive control whereas β-actin serves the loading control. **(B)** Colony formation of *METTL8* KD GSCs (n=4) (mean ± SD). ***P* <0.01, ****P* <0.001. **(C)** Representative images of colony formation in GSCs upon *METTL8* KD. **(D)** Transwell invasion assay of *METTL8* KD GSCs. Representative images shown on the right. (*n* = 3) (mean ± SD). ****P* < 0.001. **(E)** Western blot analysis of CC3 levels in *METTL8* KD GSC TS576, with or without TMZ treatment (200µM, 5 days). METTL8 and γH2AX serve as positive controls, whereas β-actin serves as a loading control.

**Supplementary Fig. S3. METTL8 mediates mt-tRNA m^3^c modification for mitochondrial translation and respiration in GSC. (A)** Volcano plot showing the proteins which were enriched in myc-METTL8 compared to myc-MTS-GFP immunoprecipitates using 293T cell lysates. **(B)** Western blot analysis of MRPS15 and MRPL13 in myc-METTL8 immunoprecipitates of 293T cell lysates, in the presence or absence of RNase A treatment. METTL8 and GFP serve as the positive controls. **(C)** Western blot analysis of MRPS15 and MRPL13 in METTL8 immunoprecipitates of GSC lysate. METTL8 serves as the positive control. **(D)** Western blot analysis of p-DRP1^S616^, p-DRP1^S637^ and DRP1 levels in GSCs upon *METTL8* KD. METTL8 and GAPDH serve as the positive and loading controls, respectively. Nocodazole (50ng/ml, 16 hrs) and forskolin (3 hrs) treatments act as controls for p-DRP1^S616^ and p-DRP1^S637^, respectively. **(E)** Comparison of *METTL8* expression in different transcriptomic subtypes of GBM using pathway-based classification. Wilcoxon-Mann-Whitney test.

**Supplementary Fig. S4. *METTL8* loss inactivates RTK signaling via HIF1α downregulation in GSC. (A)** HIF1α, H3K4me3 and H3K27ac ChIP-Seq tracks for *PDGFRA*, *ERBB3*, *TYRO3* and *EPHA7* in the indicated cell lines. **(B)** Western blot analysis of PDGFRα, ErbB3, TYRO3, EphaA7, p-Akt^S473^, and Akt levels in GSC TS576 upon gene silencing of the indicated RTK gene. The respective RTK gene and β-actin serve as positive and loading controls, respectively. **(C)** Correlative analysis of *METTL8* mRNA levels with EGFR_pY1068, EGFR_pY1173, Akt, Akt pS473, and Akt pT308 protein levels between *METTL8*^high^ vs *METTL8*^low^ gliomas (TCGA) in the RPPA dataset. **P* <0.05; ***P* <0.01; ****P* <0.0001; Wilcoxon-Mann-Whitney test.

**Supplementary Fig. S5. Proposed model of how METTL8 links mt-tRNA m^3^C modification to the HIF1α/RTK/Akt axis to sustain GBM stemness and tumorigenicity.**
