# Supplementary material for: METTL8 links mt-tRNA m3C modification to the HIF1α/RTK/Akt axis to sustain GBM stemness and tumorigenicity
Source: Cell Death Dis. 2024 May 14;15(5):338. doi: 10.1038/s41419-024-06718-2 (PMC11093979; doi:10.1038/s41419-024-06718-2)
Supplement: Supplementary file 2 — Supplementary Table [file 41419_2024_6718_MOESM2_ESM.docx]

**SUPPLEMENTARY TABLES**

**METTL8 links mt-tRNA m^3^c modification to the HIF1α/RTK/Akt axis to sustain GBM stemness and tumorigenicity**

Bernice Woon Li Lee^1,2†^, You Heng Chuah^1,2†^, Jeehyun Yoon^1,2†^, Oleg V. Grinchuk^1,2†^, Yajing Liang^1^, Jayshree L Hirpara^3^, Yating Shen^4,5^, Loo Chien Wang^6^, Yan Ting Lim^6^, Tianyun Zhao^6^, Radoslaw M Sobota^6^, Tseng Tsai Yeo^7^, Andrea Li Ann Wong^8^, Kejia Teo^7^, Vincent Diong Weng Nga^7^, Bryce Wei Quan Tan^9^, Toshio Suda^3,10^, Tan Boon Toh^4,5^, Shazib Pervaiz^1,2,11^, Zhewang Lin^12^, Derrick Sek Tong Ong^1,2,13,14^*

**Supplementary Table S1. Primers used for cloning.**

| **Primer names** | **Sequences (5’🡪3’)** |
| --- | --- |
| IKKB-SpeI-F | AAAACTAGTATGGACTACAAAGACG |
| IKKB-MluI-R | AAAACGCGTTGAGGCCTGCTCCAGGC |
| shPDGFRA#1-F | CCGGCTACTACTGTTATCAGTAATGCTCGAGCATTACTGATAACAGTAGTAGTTTTTG |
| shPDGFRA#1-R | AATTCAAAAACTACTACTGTTATCAGTAATGCTCGAGCATTACTGATAACAGTAGTAG |
| shPDGFRA#2-F | CCGGGCTAGCAATTGCGACCTTAATCTCGAGATTAAGGTCGCAATTGCTAGCTTTTTG |
| shPDGFRA#2-R | AATTCAAAAAGCTAGCAATTGCGACCTTAATCTCGAGATTAAGGTCGCAATTGCTAGC |
| shERBB3#1-F | CCGGGAATTCTCTACTCTACCATTGCTCGAGCAATGGTAGAGTAGAGAATTCTTTTTG |
| shERBB3#1-R | AATTCAAAAAGAATTCTCTACTCTACCATTGCTCGAGCAATGGTAGAGTAGAGAATTC |
| shERBB3#2-F | CCGGCTTCGTCATGTTGAACTATAACTCGAGTTATAGTTCAACATGACGAAGTTTTTG |
| shERBB3#2-R | AATTCAAAAACTTCGTCATGTTGAACTATAACTCGAGTTATAGTTCAACATGACGAAG |
| shTYRO3#1-F | CCGGCCAGTGACTGTCGGTACATACCTCGAGGTATGTACCGACAGTCACTGGTTTTTG |
| shTYRO3#1-R | AATTCAAAAACCAGTGACTGTCGGTACATACCTCGAGGTATGTACCGACAGTCACTGG |
| shTYRO3#2-F | CCGGTTGGTATCTCAGGTCTGAATCCTCGAGGATTCAGACCTGAGATACCAATTTTTG |
| shTYRO3#2-R | AATTCAAAAATTGGTATCTCAGGTCTGAATCCTCGAGGATTCAGACCTGAGATACCAA |
| shEPHA7#1-F | CCGGCGATGTGACCTACAGAATATTCTCGAGAATATTCTGTAGGTCACATCGTTTTTG |
| shEPHA7#1-R | AATTCAAAAACGATGTGACCTACAGAATATTCTCGAGAATATTCTGTAGGTCACATCG |
| shEPHA7#2-F | CCGGGTCTACTTCAGCCTCCATTAACTCGAGTTAATGGAGGCTGAAGTAGACTTTTTG |
| shEPHA7#2-R | AATTCAAAAAGTCTACTTCAGCCTCCATTAACTCGAGTTAATGGAGGCTGAAGTAGAC |

Supplementary Table S2. RT-qPCR primers used in this study.

| **Primer names** | **Sequences (5’🡪3’)** |
| --- | --- |
| *HSP70*-F | ATGTCGGTGGTGGGCATAGA |
| *HSP70*-R | CACAGCGACGTAGCAGCTCT |
| *METTL8*-F | GGGATCACATGCAGTGGTCTA |
| *METTL8*-R | CCCTCAACAGCCAATTACGAT |
| *HIF1A*-F | TATGAGCCAGAAGAACTTTTAGGC |
| *HIF1A*-R | CACCTCTTTTGGCAAGCATCCTG |

Supplementary Table S3. Antibodies used in this study.

| **Proteins** | **Source** | **Catalogue number** |
| --- | --- | --- |
| METTL8 | GenScript | clone #1857 |
| pan-H2AZ | abcam | ab4174 |
| HIF1α | Santa Cruz | sc-13515 |
| SOD1 | BD biosciences | AB_396385 |
| TOM20 | Cell Signaling | 42460 |
| MRPS15 | Proteintech | 17006-1-AP |
| MRPL13 | Proteintech | 16241-1-AP |
| Myc tag | Cell Signaling | 2276 |
| Puromycin | Sigma | MABE343 |
| HSP60 | BD biosciences | AB_399008 |
| GFAP | Biolegend | 644702 |
| OLIG2 | Millipore | AB9610 |
| PDGFRα | Cell Signaling | 3174 |
| ERBB3 | Cell Signaling | 12708 |
| TYRO3 | Santa Cruz | sc-166359 |
| EphA7 | Santa Cruz | sc-393973 |
| p-AkT (Ser473) | Cell Signaling | 4060 |
| AkT | Cell Signaling | 4691 |
| p-STAT3 (Y705) | Cell Signaling | 9145 |
| STAT3 | Cell Signaling | 9139 |
| PTEN | Cell Signaling | 9559 |
| p-AMPK | Cell Signaling | 2535 |
| AMPK | Cell Signaling | 2532 |
| FLAG-M2 | Sigma | F1804 |
| CD44 | Cell Signaling | 37259 |
| p-p65 (S536) | Cell Signaling | 3033T |
| p65 | Cell Signaling | 8242 |
| γH2AX | Millipore | 05-636 |
| PLK1 | Cell Signaling | 4513 |
| Cleaved Caspase 3 (CC3) | Cell Signaling | 9661 |
| β-actin | Sigma | A5316 |
| Vinculin | Sigma | V9171 |
| p-DRP1 (S616) | Cell Signaling | 4494 |
| p-DRP1 (S637) | Cell Signaling | 6319 |
| DRP1 | Cell Signaling | 5391 |

**Supplementary Table S4. ChIP-qPCR primers used in this study.**

| **Primer names** | **Sequences (5’🡪3’)** |
| --- | --- |
| METTL8-ChIP-F | GGGATAGGTGGTAATAGGGCC |
| METTL8-ChIP-R | TCCACTCTGCCCTCATGTTT |
| PDGFRA-ChIP-F | TCAACTGAGGTCACCACGAA |
| PDGFRA-ChIP-R | CTGAGCTCCACTACCCCAAA |
| ERBB3-ChIP-F | CCTTGGCCTATCTCCTGCAG |
| ERBB3-ChIP-R | CGAGGCTGGAGTAGGGATTG |
| TYRO3-ChIP-F | GGGATGCTGGATTCTGGGAT |
| TYRO3-ChIP-R | CCCCTCCTTTCCCTAAACCC |
| EPHA7-ChIP-F | CAGAGGAGAACGAGGATGGT |
| EPHA7-ChIP-R | AGAGTGTAAGTTGGGAGCGT |
