# Supplementary figures and images for: METTL8 links mt-tRNA m3C modification to the HIF1α/RTK/Akt axis to sustain GBM stemness and tumorigenicity
Source: Cell Death Dis. 2024 May 14;15(5):338. doi: 10.1038/s41419-024-06718-2 (PMC11093979; doi:10.1038/s41419-024-06718-2)

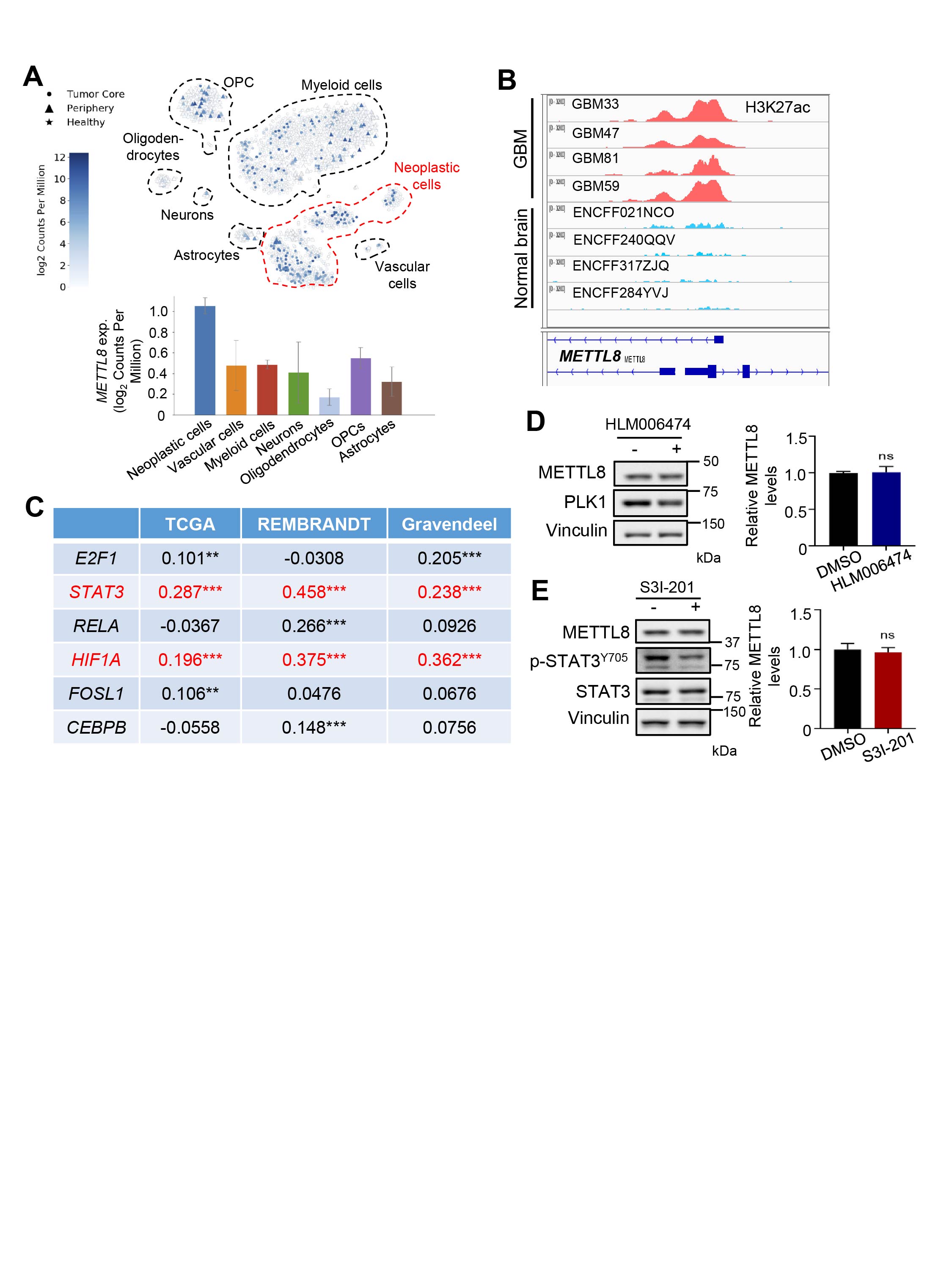

Supplement: Supplementary file 3 — Figure S1 [file 41419_2024_6718_MOESM3_ESM.jpg]

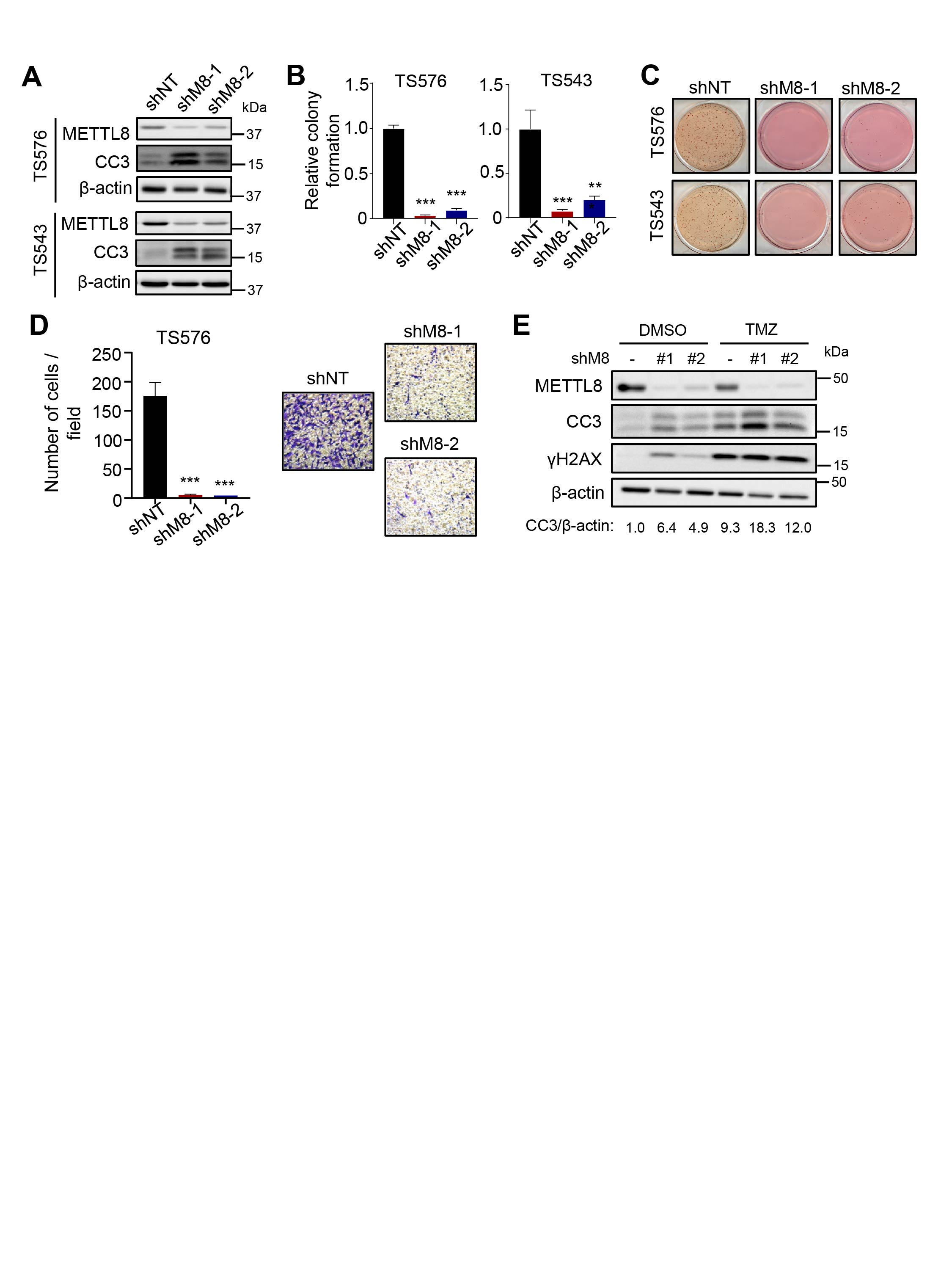

Supplement: Supplementary file 4 — Figure S2 [file 41419_2024_6718_MOESM4_ESM.jpg]

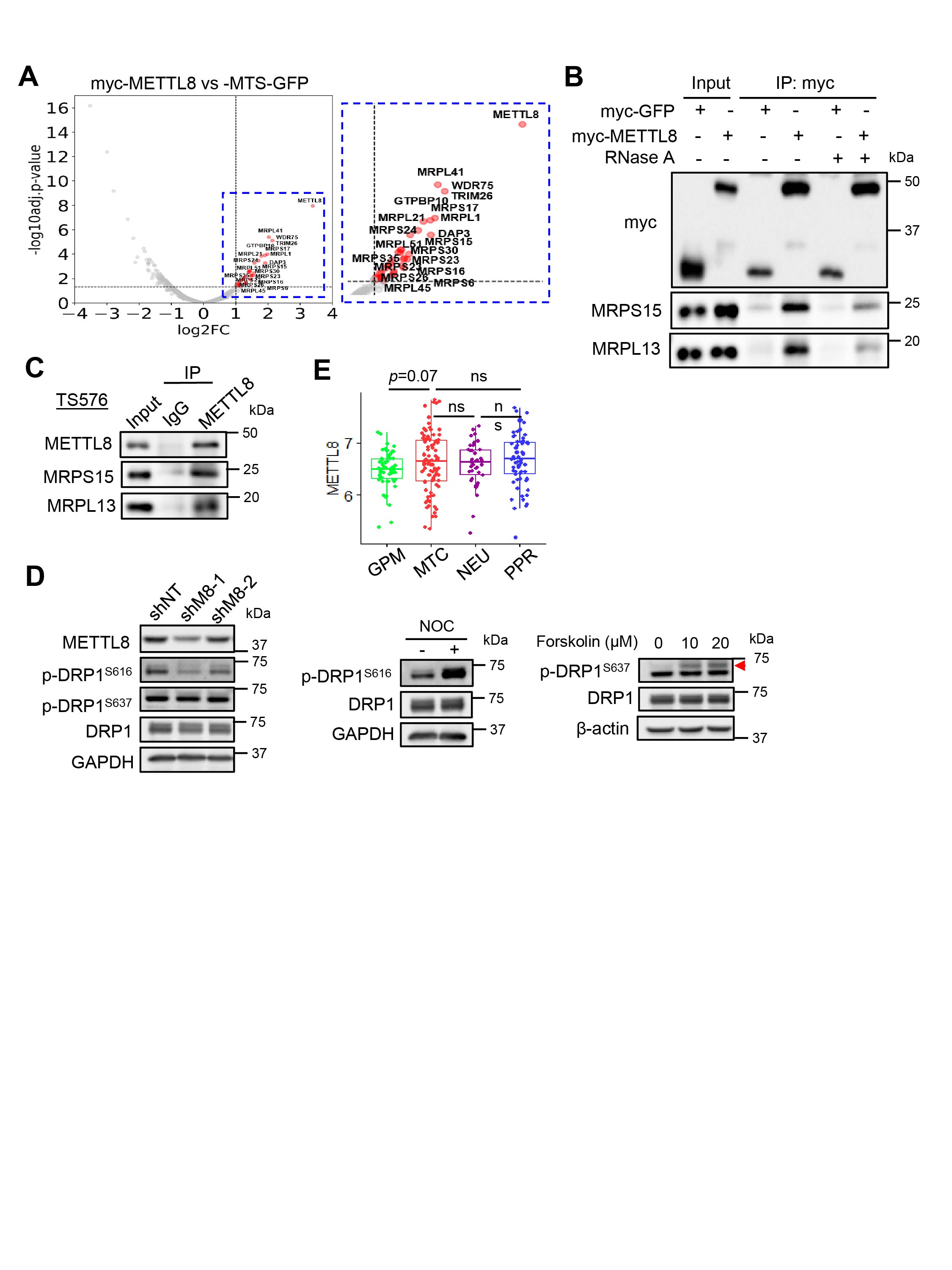

Supplement: Supplementary file 5 — Figure S3 [file 41419_2024_6718_MOESM5_ESM.jpg]

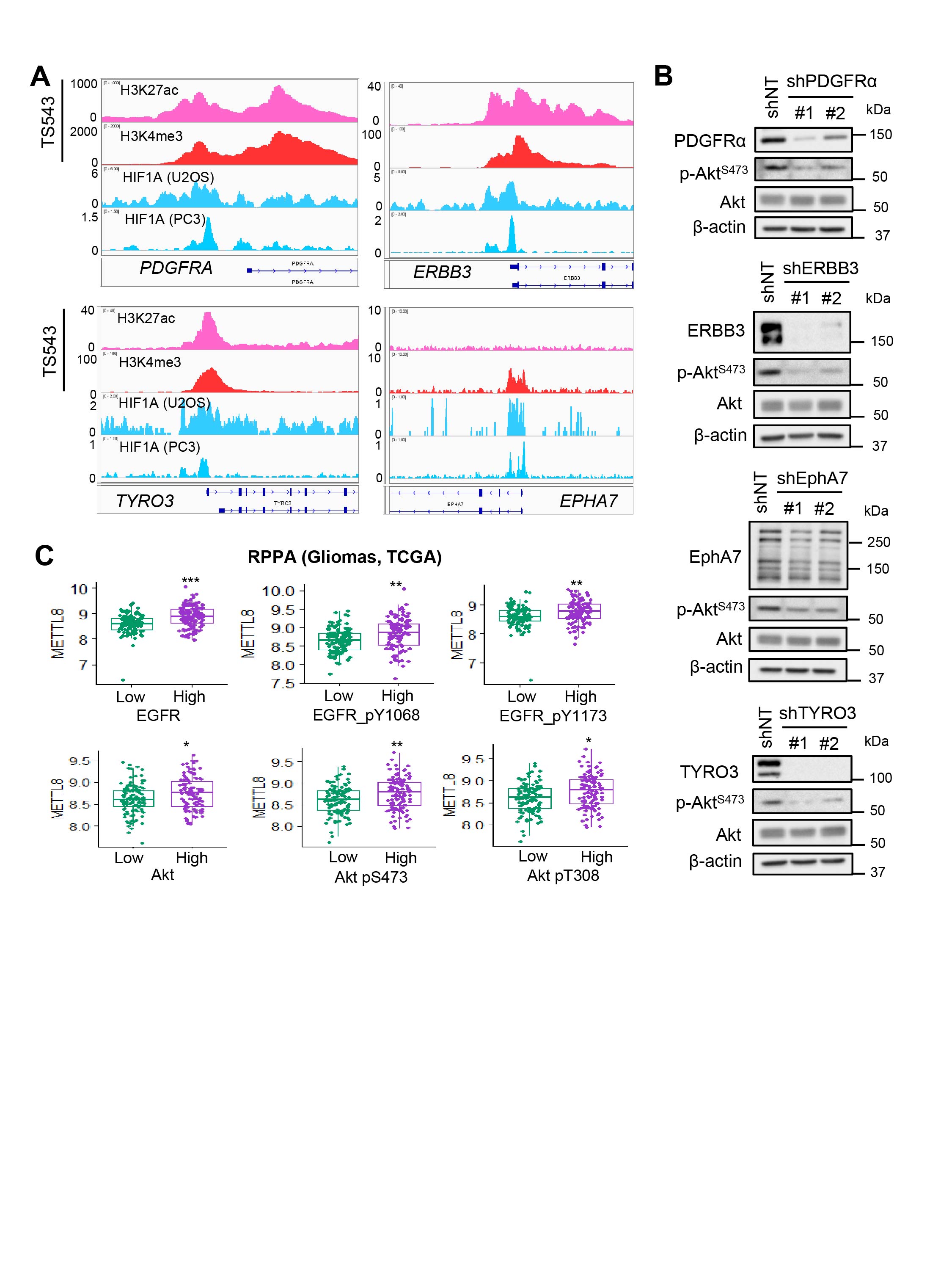

Supplement: Supplementary file 6 — Figure S4 [file 41419_2024_6718_MOESM6_ESM.jpg]

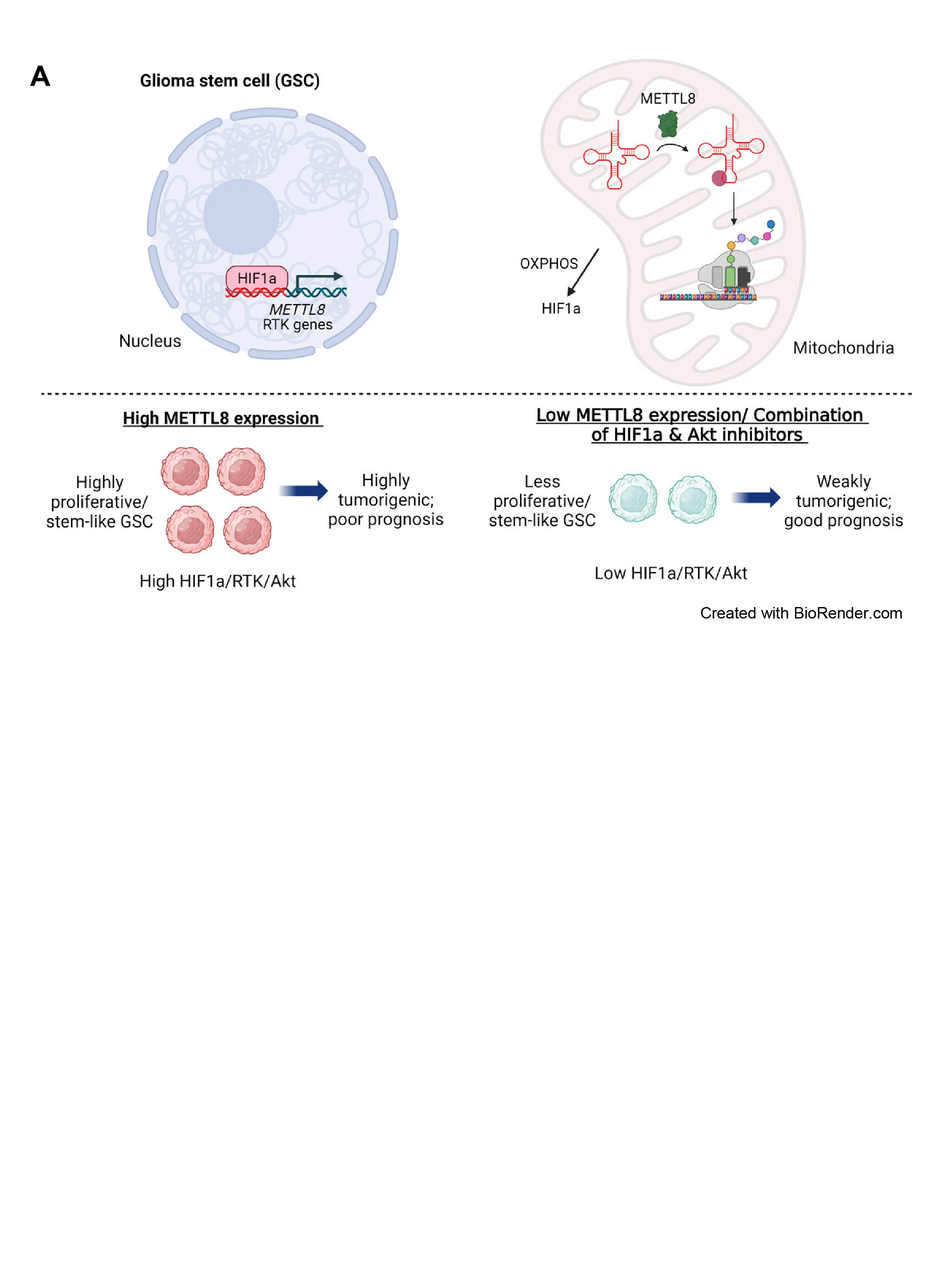

Supplement: Supplementary file 7 — Figure S5 [file 41419_2024_6718_MOESM7_ESM.jpg]

Fig. S1D

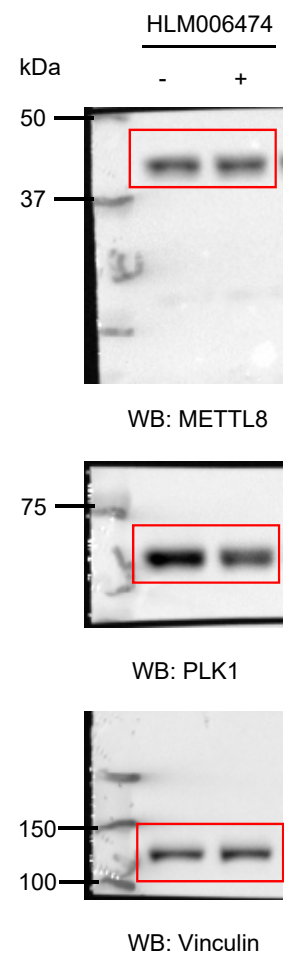

Fig. S1E

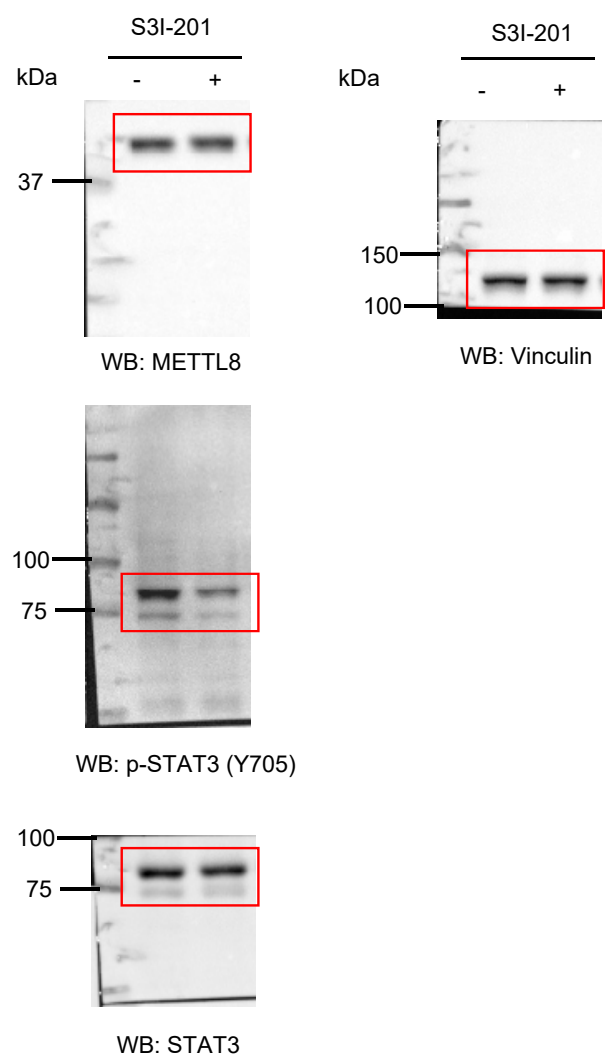

Fig S2A

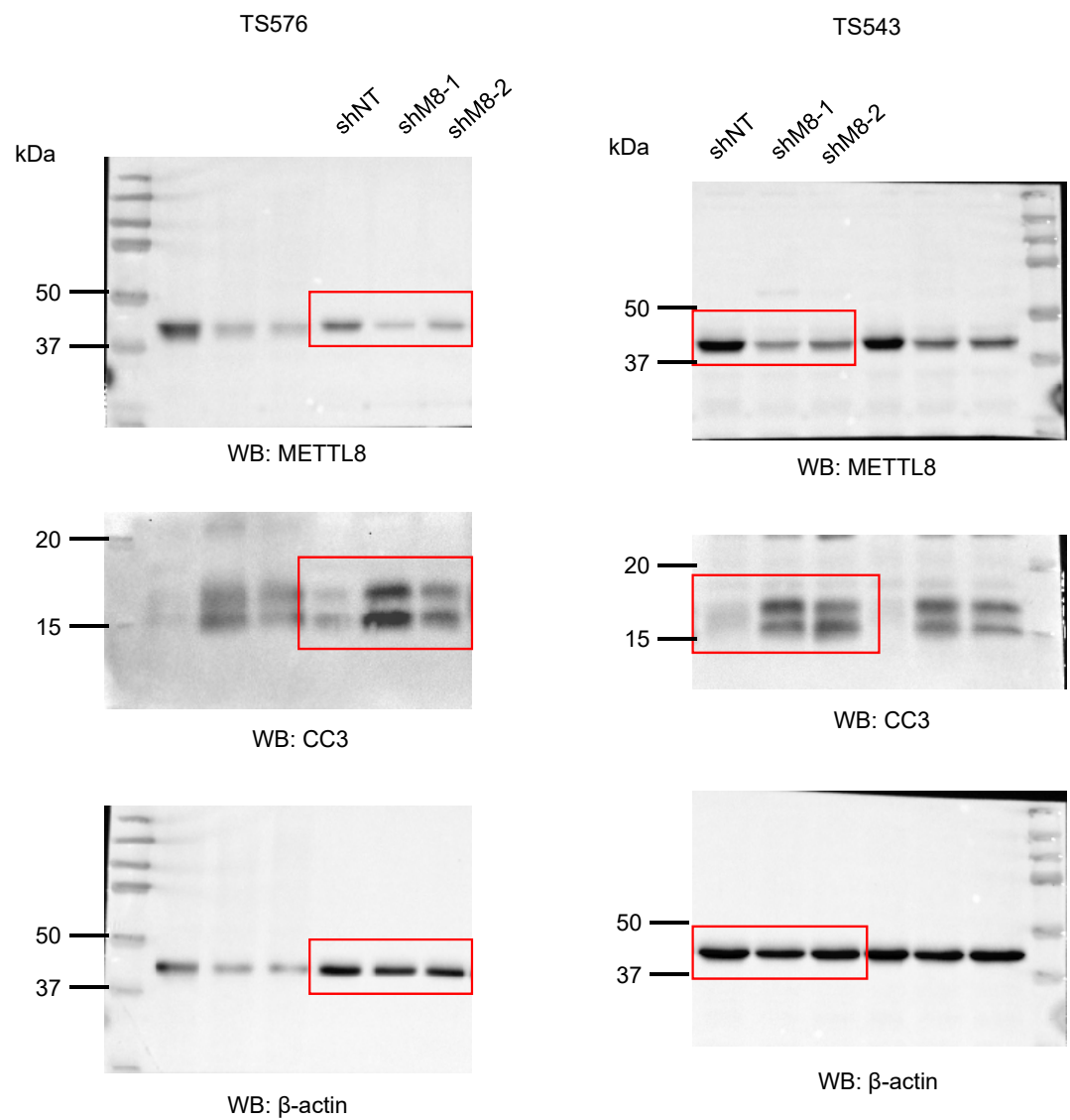

Fig S2E

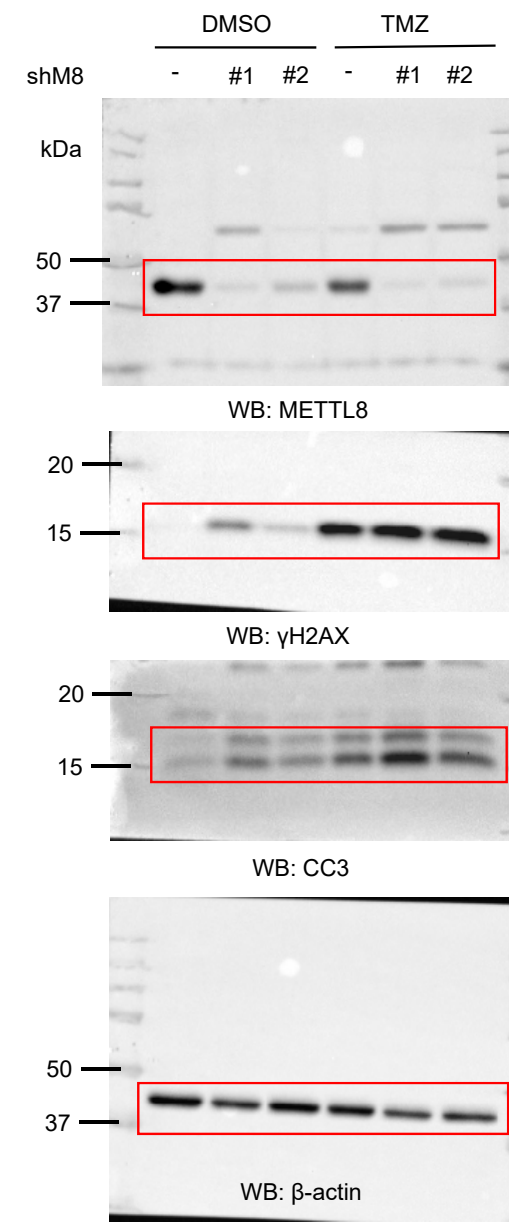

Fig. S3B

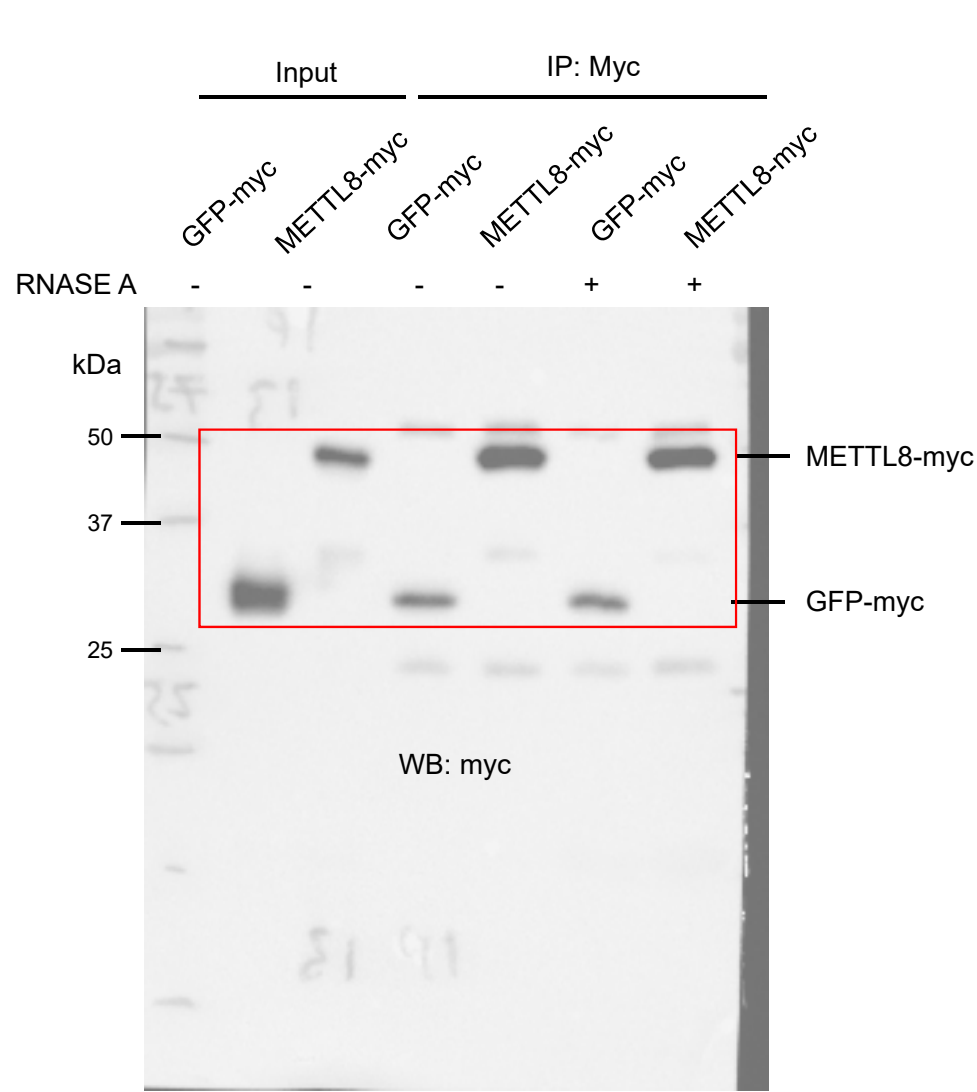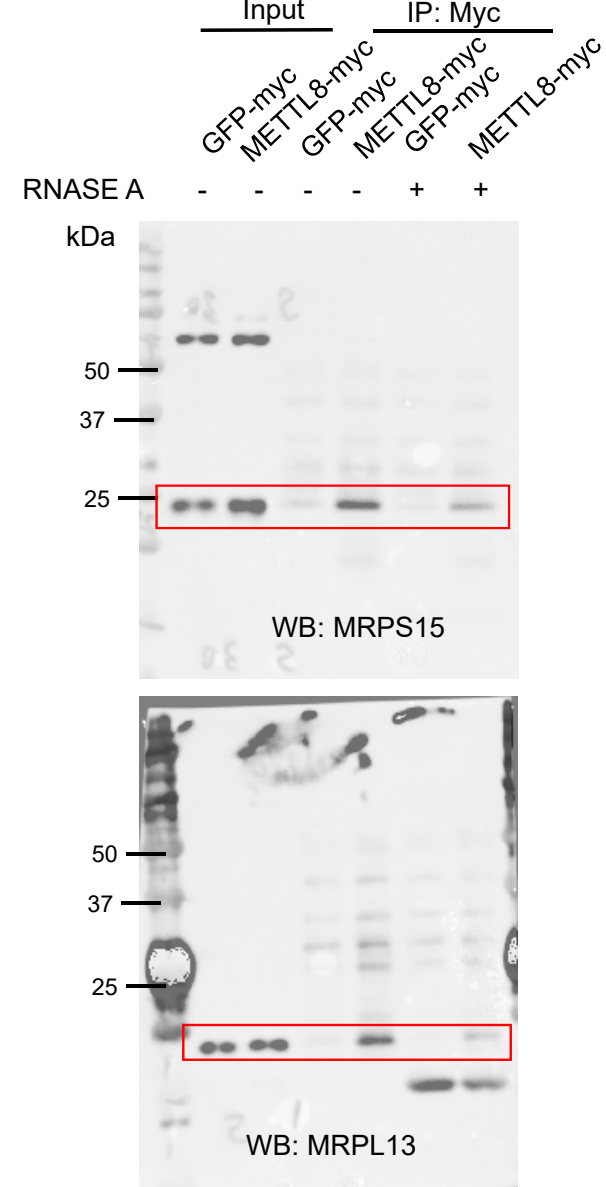

Fig. S3C

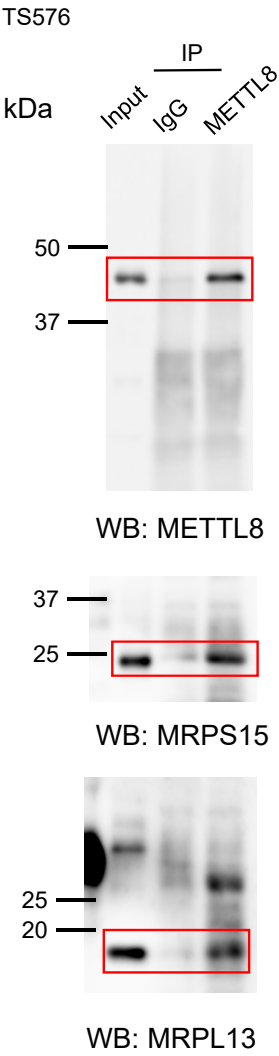

**Fig. S3D**

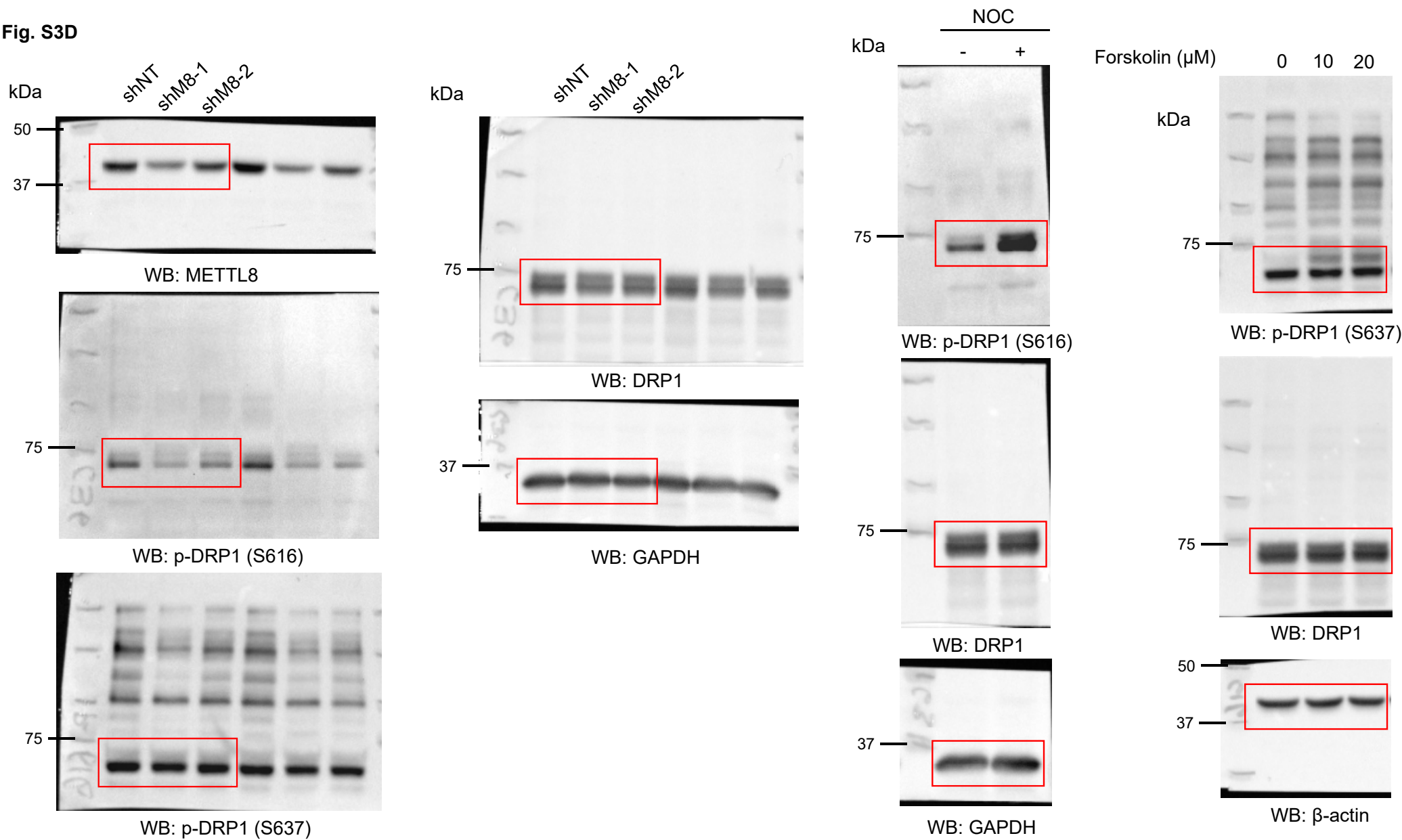

**Fig. S4B**

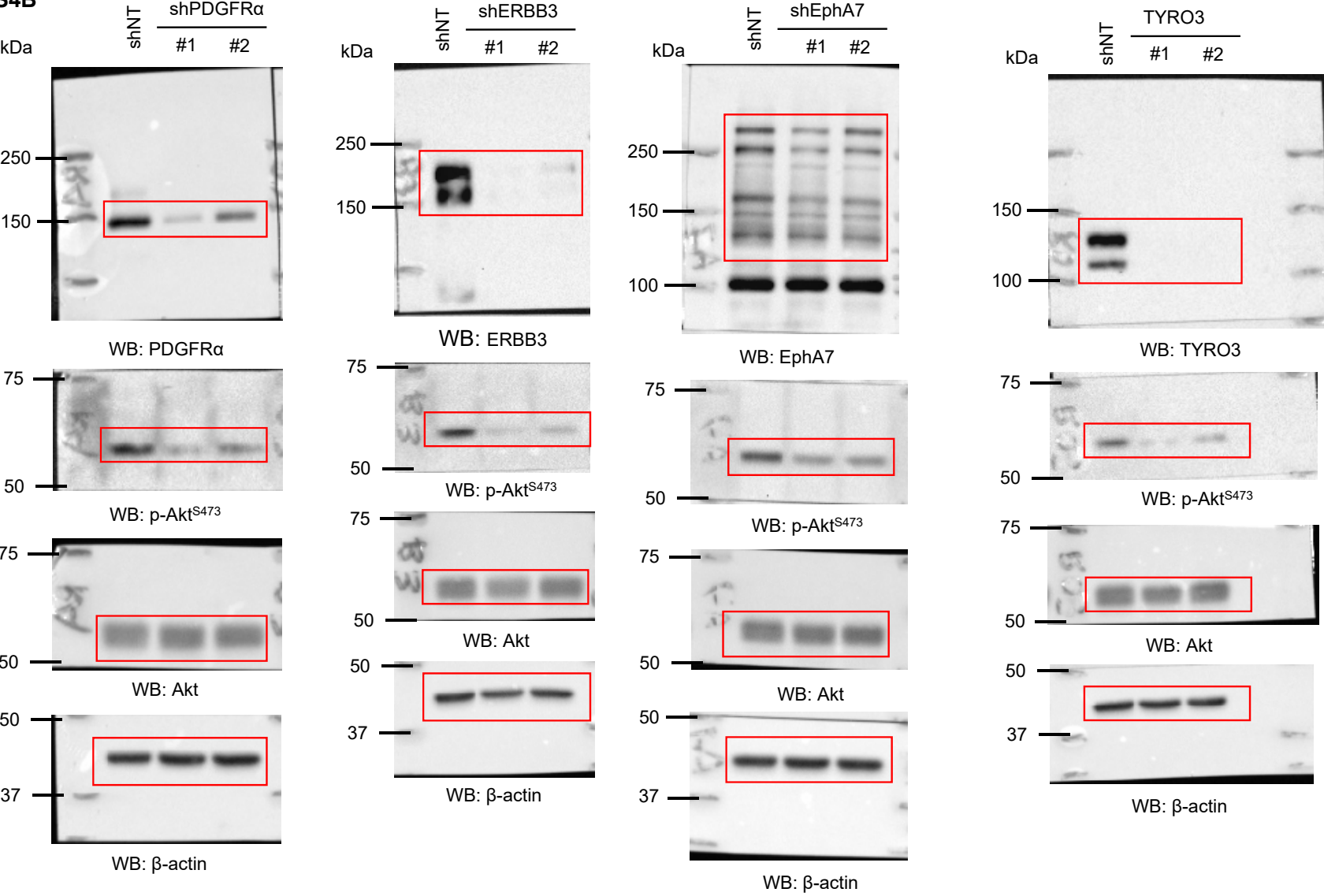

Supplement: Supplementary file 9 — Source data for Supplementary Figures [file 41419_2024_6718_MOESM9_ESM.pdf]
