# Supplementary material for: METTL8 links mt-tRNA m3C modification to the HIF1α/RTK/Akt axis to sustain GBM stemness and tumorigenicity
Source: Cell Death Dis. 2024 May 14;15(5):338. doi: 10.1038/s41419-024-06718-2 (PMC11093979; doi:10.1038/s41419-024-06718-2)
Supplement: Supplementary file 8 — Source data for Figures [file 41419_2024_6718_MOESM8_ESM.pdf]

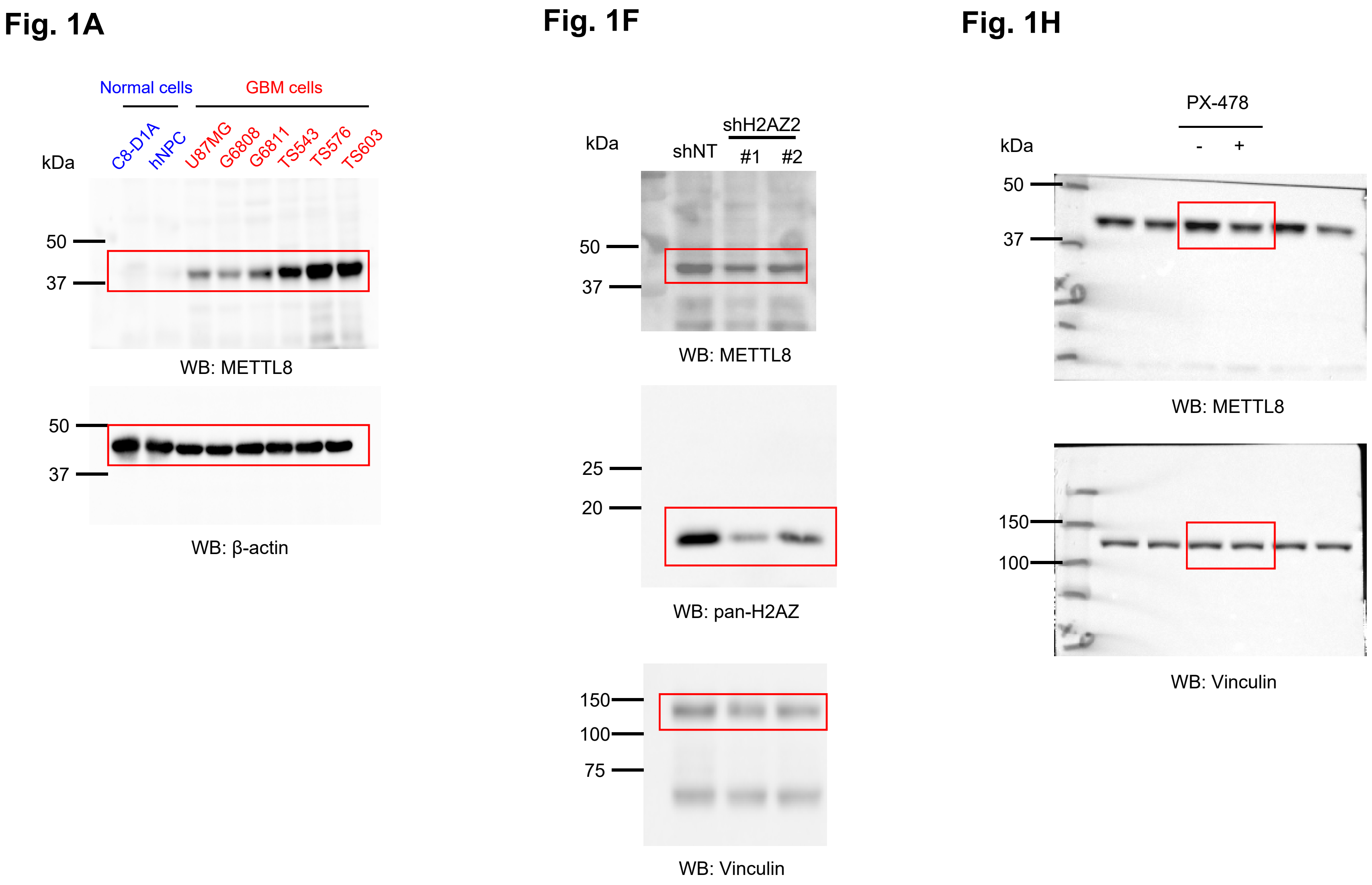

Fig. 1A
Normal cells
GBM cells
kDa
C8-D1A
hNPC
U87MG
G6808
G6811
TS543
TS576
TS603
50
37
WB: METTL8
50
37
WB: β-actin
Fig. 1F
kDa
shNT
shH2AZ2
#1
#2
50
37
WB: METTL8
25
20
WB: pan-H2AZ
150
100
75
WB: Vinculin
Fig. 1H
PX-478
kDa
-
+
50
37
WB: METTL8
150
100
WB: Vinculin

**Fig. 2E**

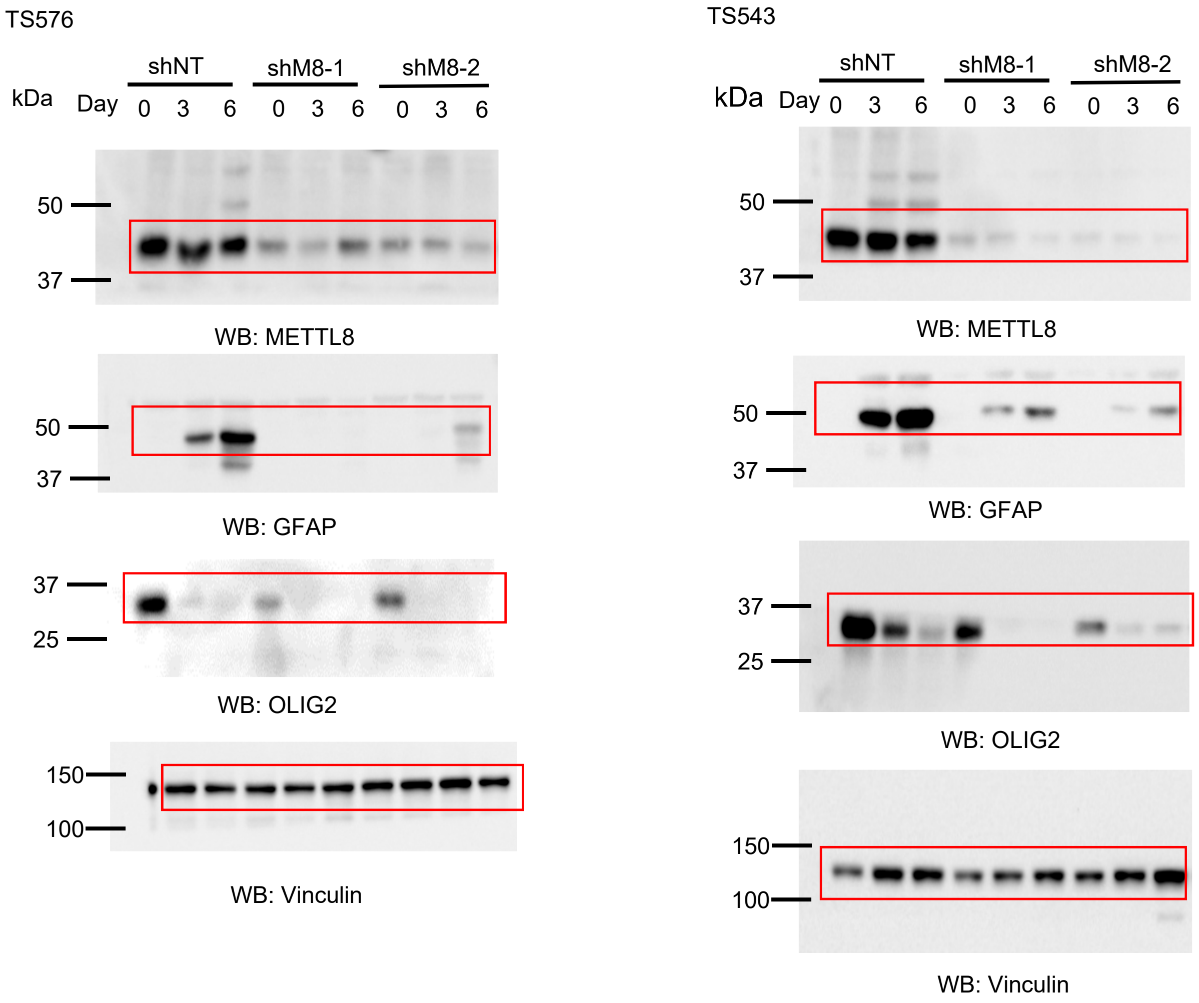
TS576
shNT
shM8-1
shM8-2
kDa
Day 0 3 6 0 3 6 0 3 6
50
37
WB: METTL8
50
37
WB: GFAP
37
25
WB: OLIG2
150
100
WB: Vinculin
TS543
shNT
shM8-1
shM8-2
kDa
Day 0 3 6 0 3 6 0 3 6
50
37
WB: METTL8
50
37
WB: GFAP
37
25
WB: OLIG2
150
100
WB: Vinculin

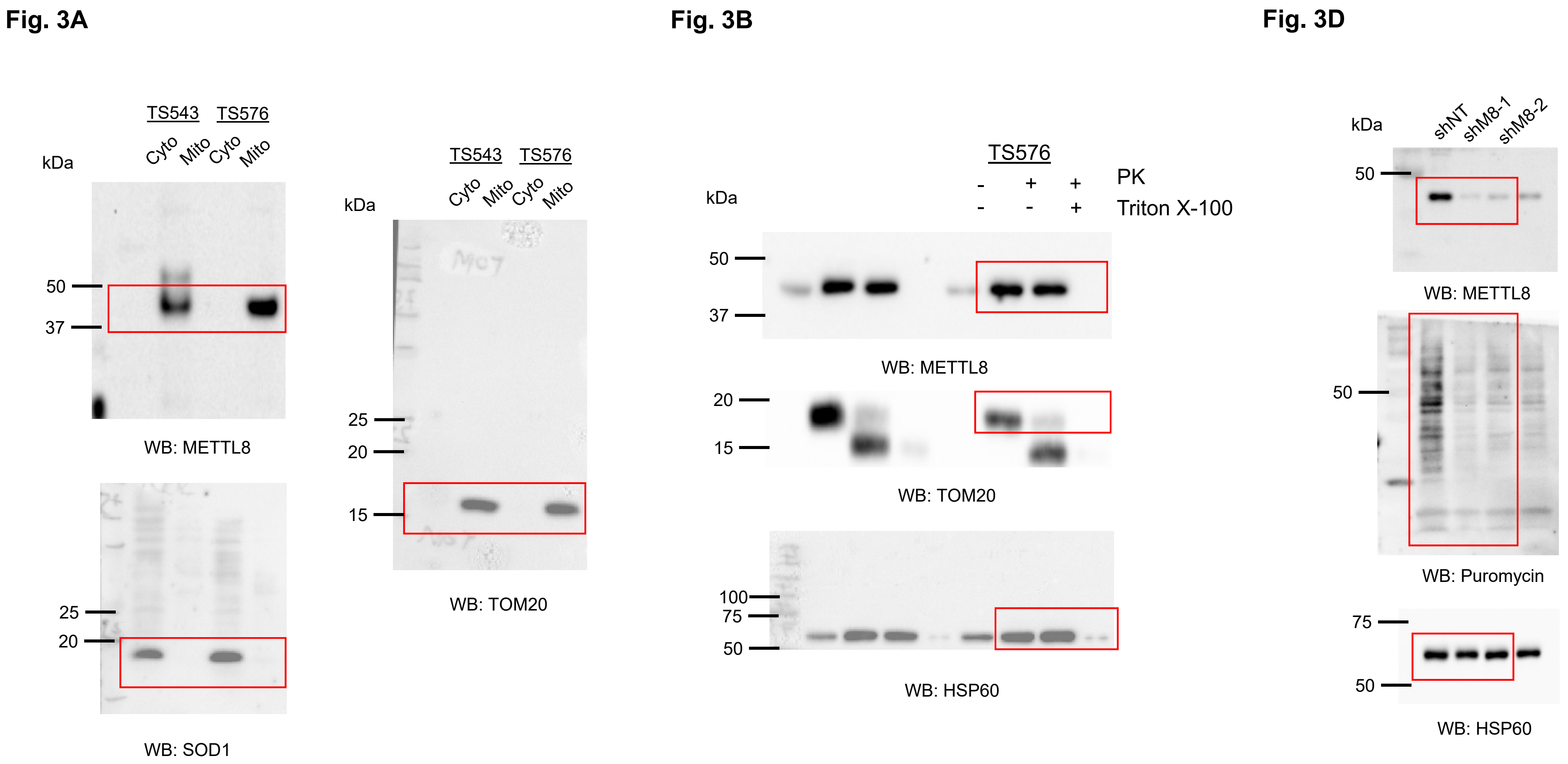
Fig. 3A
TS543
TS576
Cyto
Mito
Cyto
Mito
kDa
50
37
WB: METTL8
25
20
WB: SOD1
TS543
TS576
Cyto
Mito
Cyto
Mito
kDa
25
20
15
WB: TOM20
Fig. 3B
TS576
PK
Triton X-100
- + +
- - +
kDa
50
37
WB: METTL8
20
15
WB: TOM20
100
75
50
WB: HSP60
Fig. 3D
kDa
shNT
shM8-1
shM8-2
50
WB: METTL8
50
WB: Puromycin
75
50
WB: HSP60

**Fig. 3E**

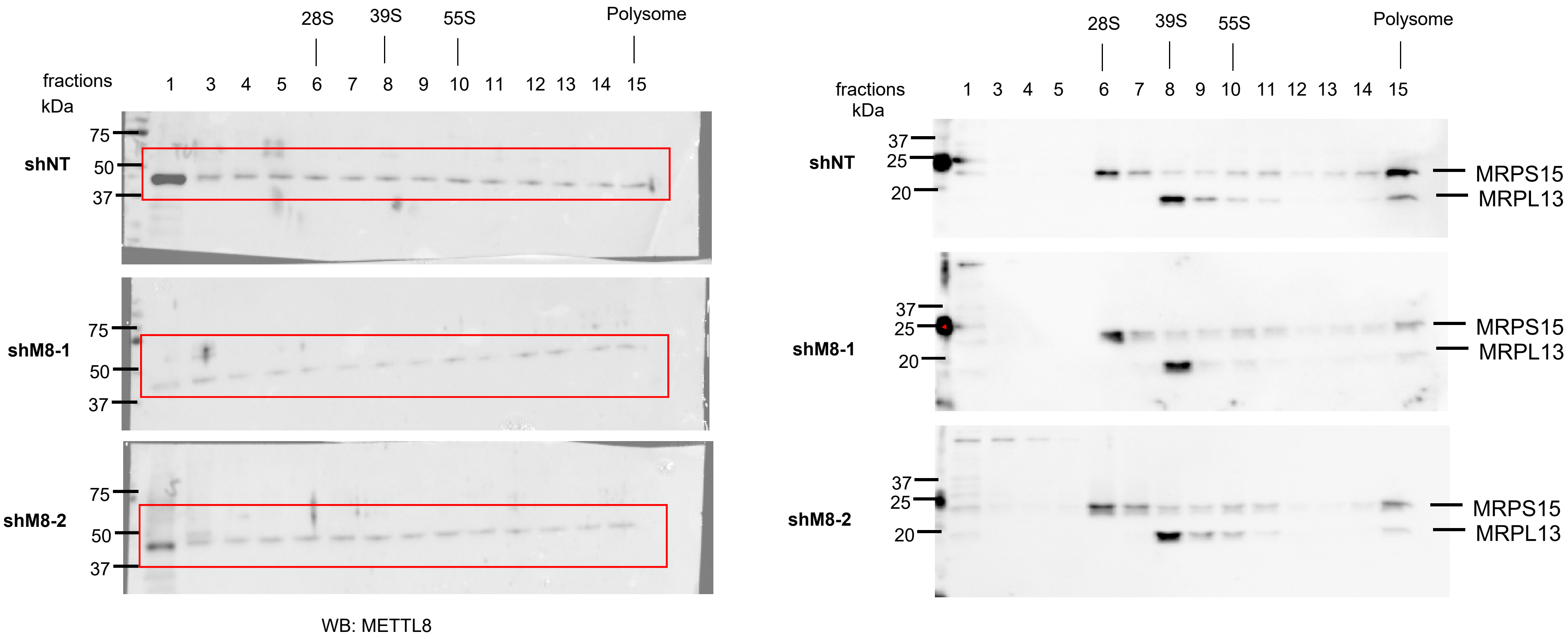

28S
39S
55S
Polysome
fractions
kDa
1 3 4 5 6 7 8 9 10 11 12 13 14 15
shNT
shM8-1
shM8-2
75
50
37
WB: METTL8
28S
39S
55S
Polysome
fractions
kDa
1 3 4 5 6 7 8 9 10 11 12 13 14 15
37
25
20
MRPS15
MRPL13

**Fig. 3H**

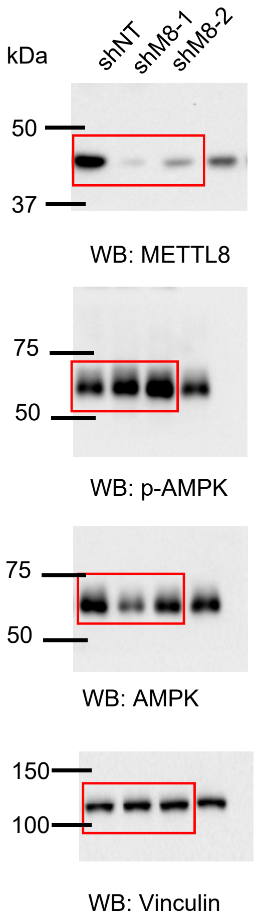
kDa
shNT
shM8-1
shM8-2
50
37
WB: METTL8
75
50
WB: p-AMPK
75
50
WB: AMPK
150
100
WB: Vinculin

Fig. 4C

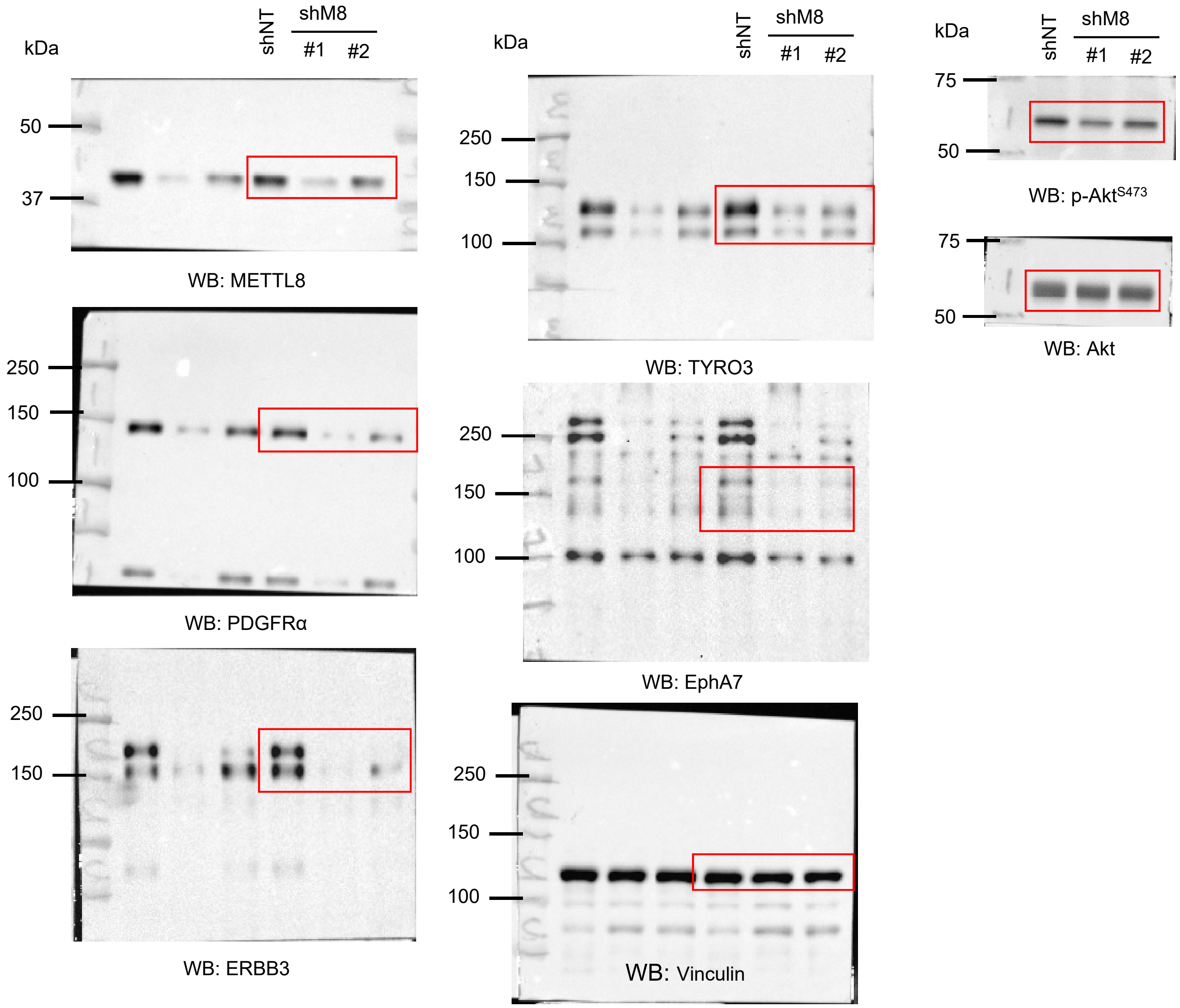
kDa
shNT
shM8
#1
#2
50
37
WB: METTL8
250
150
100
WB: PDGFRα
250
150
WB: ERBB3
250
150
100
WB: TYRO3
250
150
100
WB: EphA7
250
150
100
WB: Vinculin
75
50
WB: p-AktS473
75
50
WB: Akt

Fig. 4E

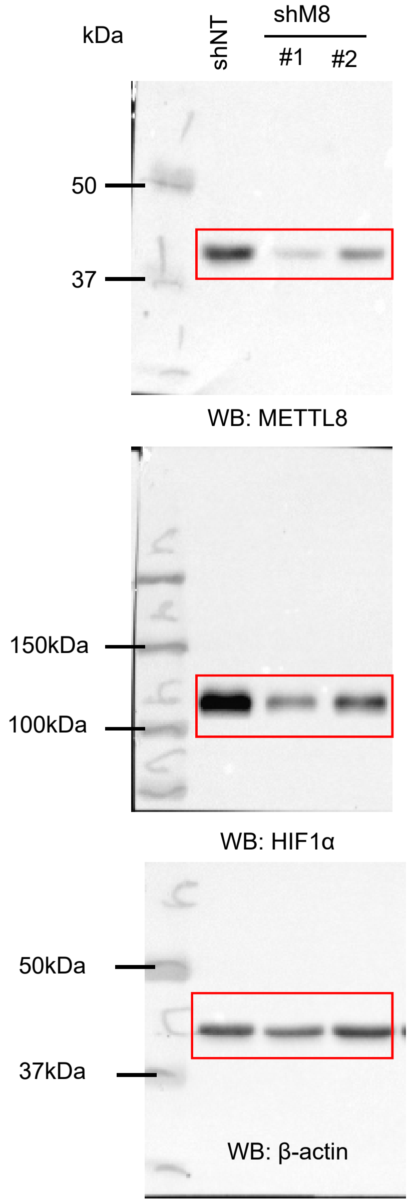
kDa
shNT
shM8
#1
#2
50
37
WB: METTL8
150kDa
100kDa
WB: HIF1α
50kDa
37kDa
WB: β-actin

**Fig. 4J**

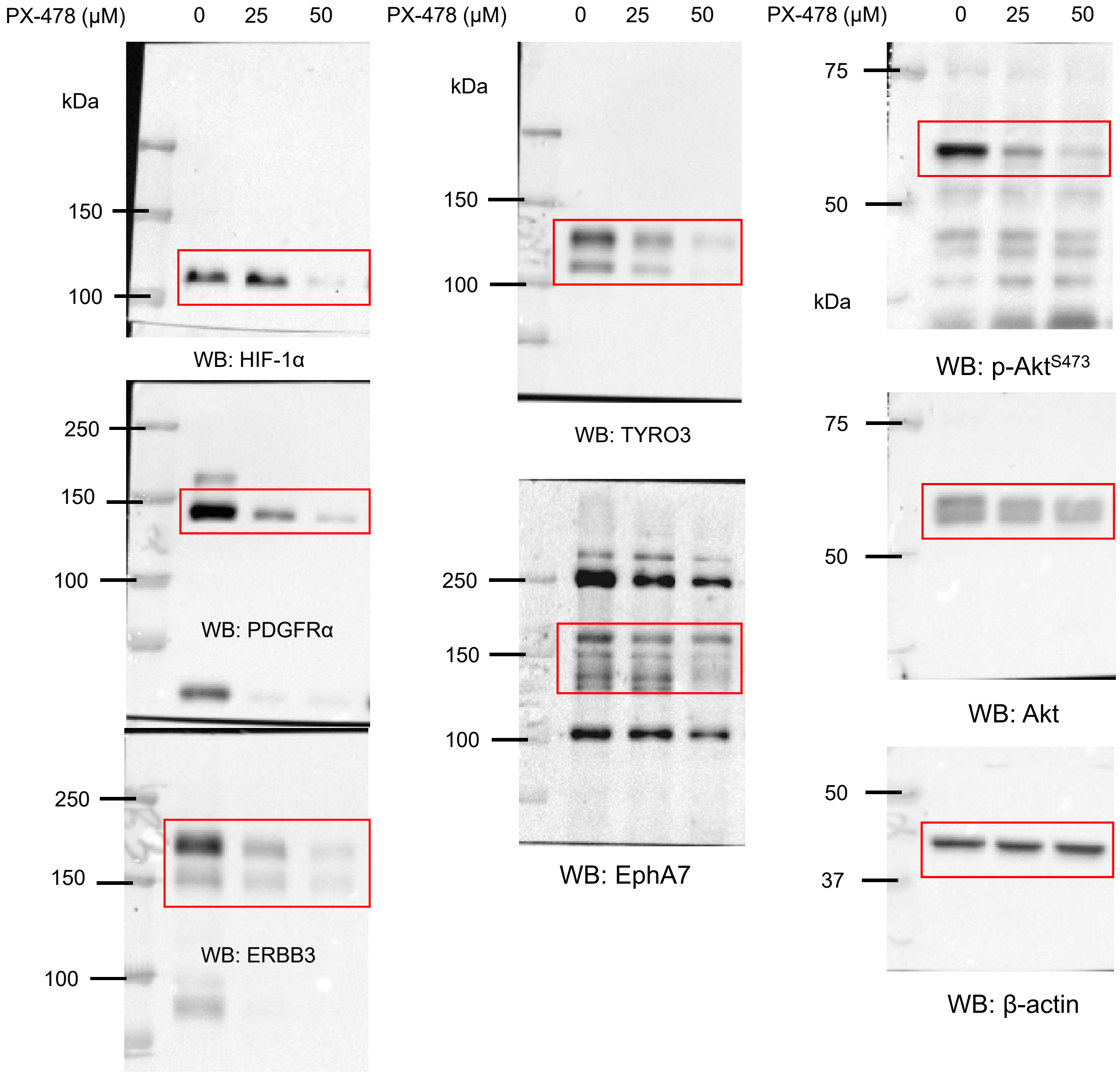

PX-478 (μM)
0
25
50
kDa
150
100
WB: HIF-1α
250
150
100
WB: PDGFRα
250
150
100
WB: ERBB3
PX-478 (μM)
0
25
50
kDa
150
100
WB: TYRO3
250
150
100
WB: EphA7
PX-478 (μM)
0
25
50
75
50
kDa
WB: p-Akt S473
75
50
WB: Akt
50
37
WB: β-actin

## Fig. 5B

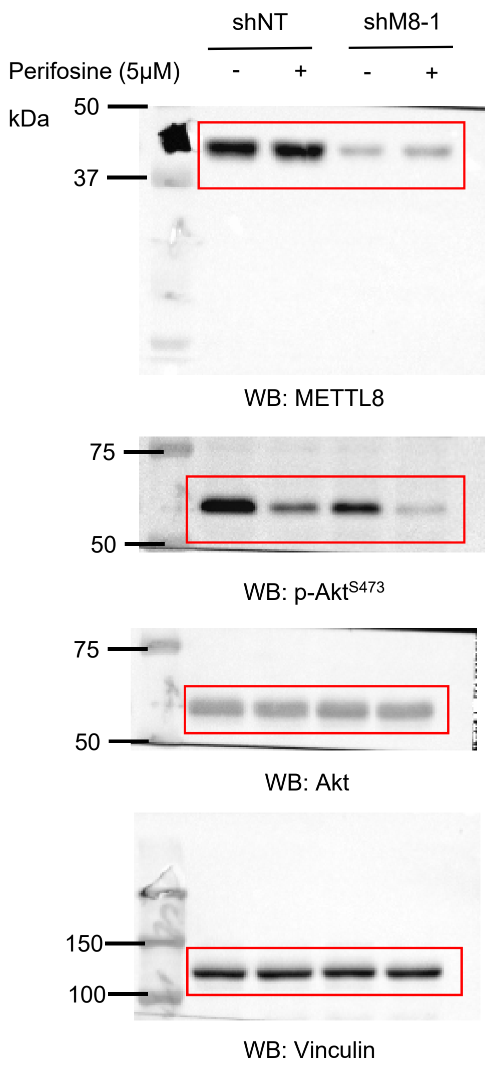

shNT
shM8-1
Perifosine (5μM)
-
+
-
+
kDa
50
37
WB: METTL8
75
50
WB: p-AktS473
75
50
WB: Akt
150
100
WB: Vinculin


## Fig. 5E

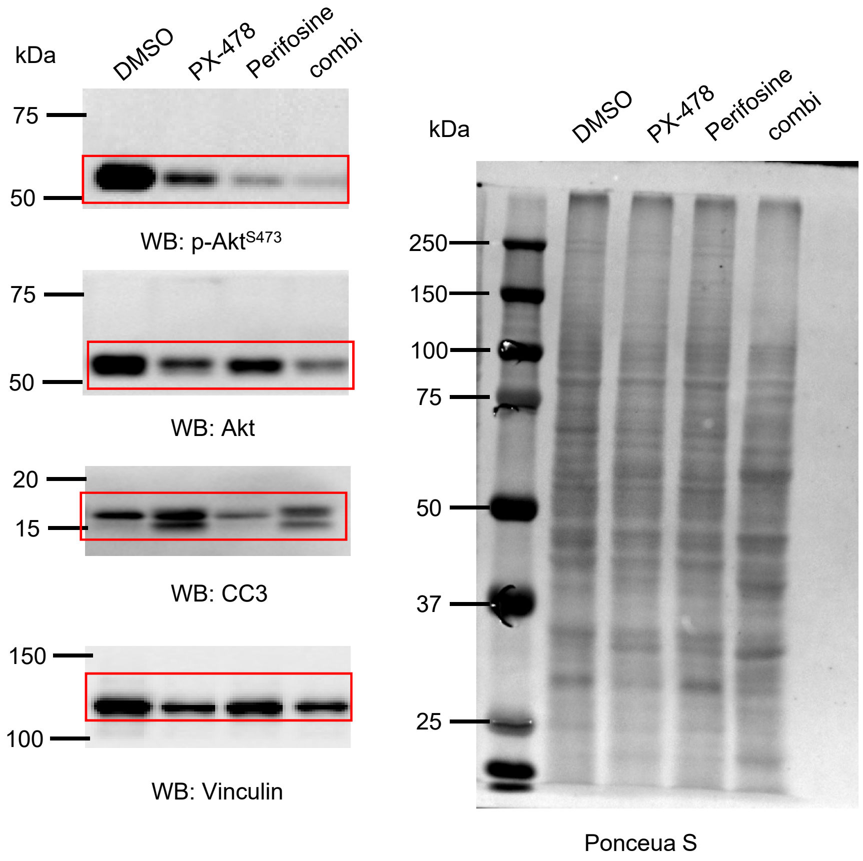

kDa
DMSO
PX-478
Perifosine
combi
75
50
WB: p-AktS473
75
50
WB: Akt
20
15
WB: CC3
150
100
WB: Vinculin
kDa
DMSO
PX-478
Perifosine
combi
250
150
100
75
50
37
25
Ponceua S

**Fig. 6A**

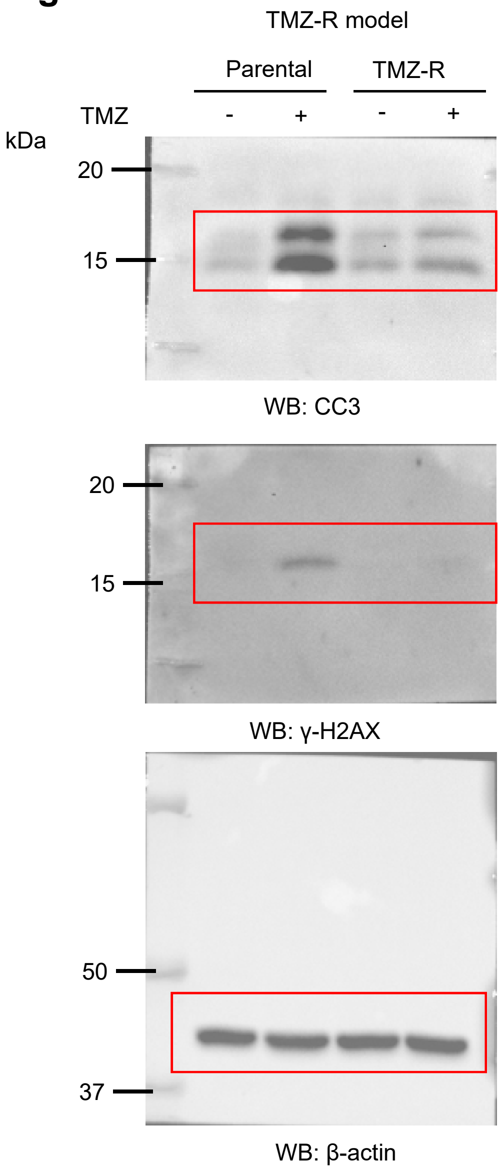
TMZ-R model
Parental
TMZ-R
TMZ
-
+
-
+
kDa
20
15
WB: CC3
20
15
WB: γ-H2AX
50
37
WB: β-actin

**Fig. 6B**

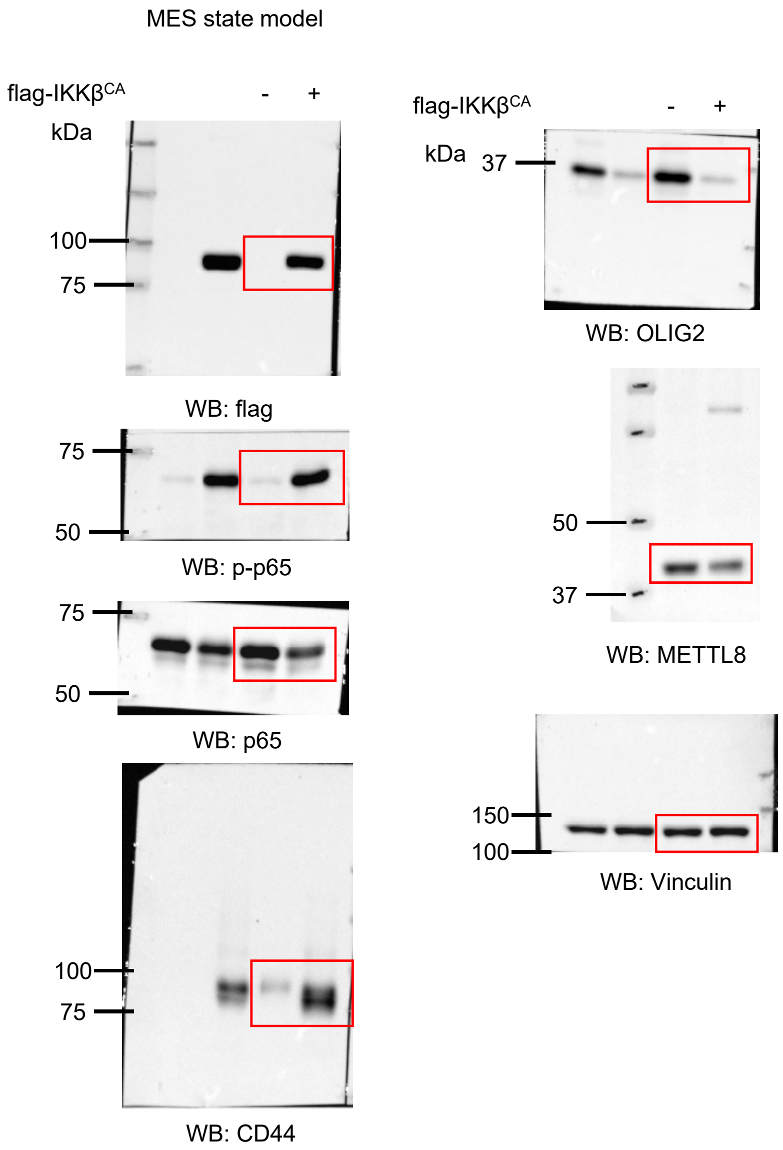
MES state model
flag-IKKβCA
-
+
kDa
100
75
WB: flag
75
50
WB: p-p65
75
50
WB: p65
100
75
WB: CD44
flag-IKKβCA
-
+
kDa
37
WB: OLIG2
50
37
WB: METTL8
150
100
WB: Vinculin

# Fig. 6E

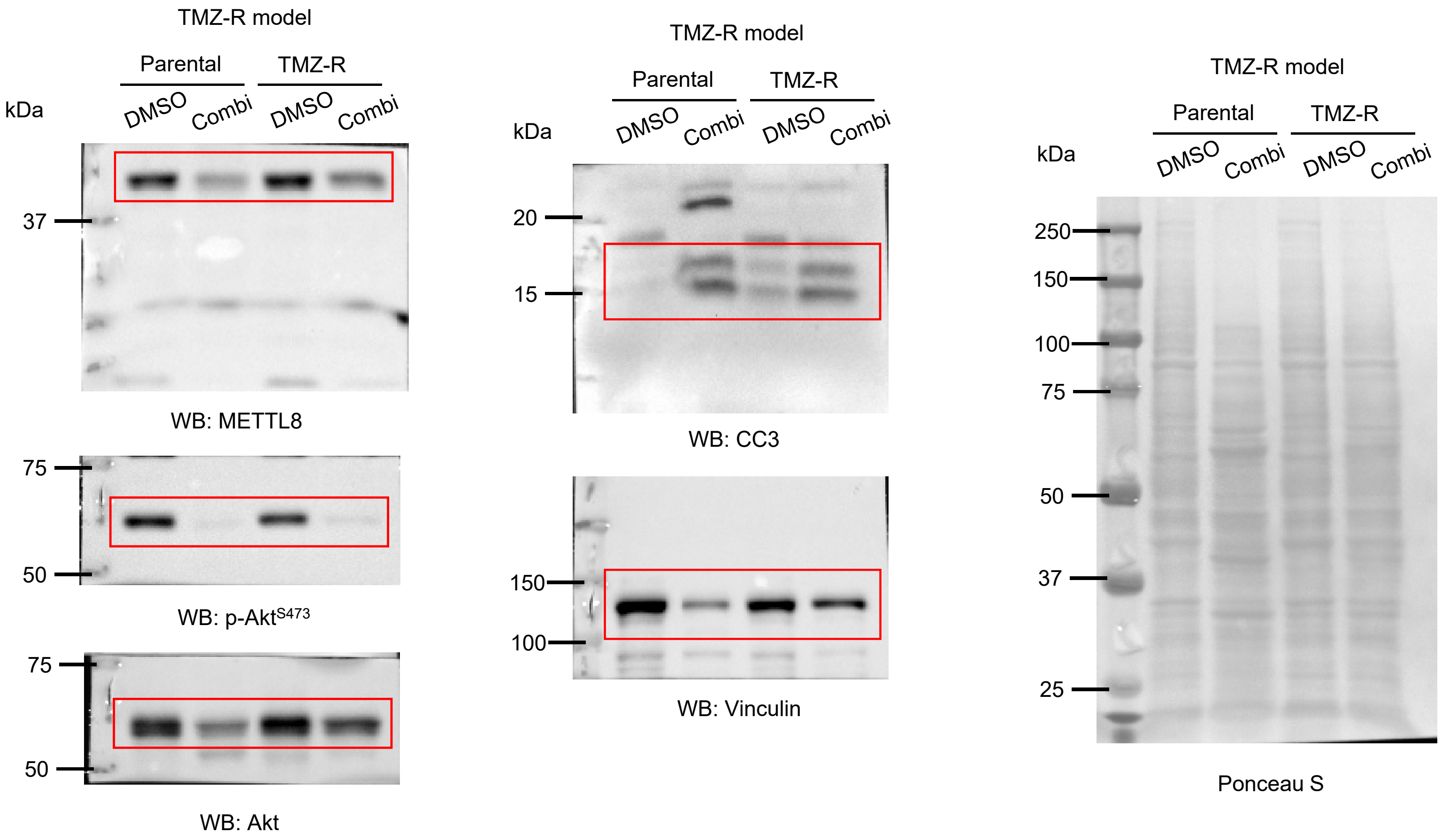

TMZ-R model
Parental
TMZ-R
kDa
DMSO
Combi
DMSO
Combi
37
WB: METTL8
75
50
WB: p-Akt$^{S473}$
75
50
WB: Akt
TMZ-R model
Parental
TMZ-R
kDa
DMSO
Combi
DMSO
Combi
20
15
WB: CC3
150
100
WB: Vinculin
TMZ-R model
Parental
TMZ-R
kDa
DMSO
Combi
DMSO
Combi
250
150
100
75
50
37
25
Ponceau S

**Fig. 6E**

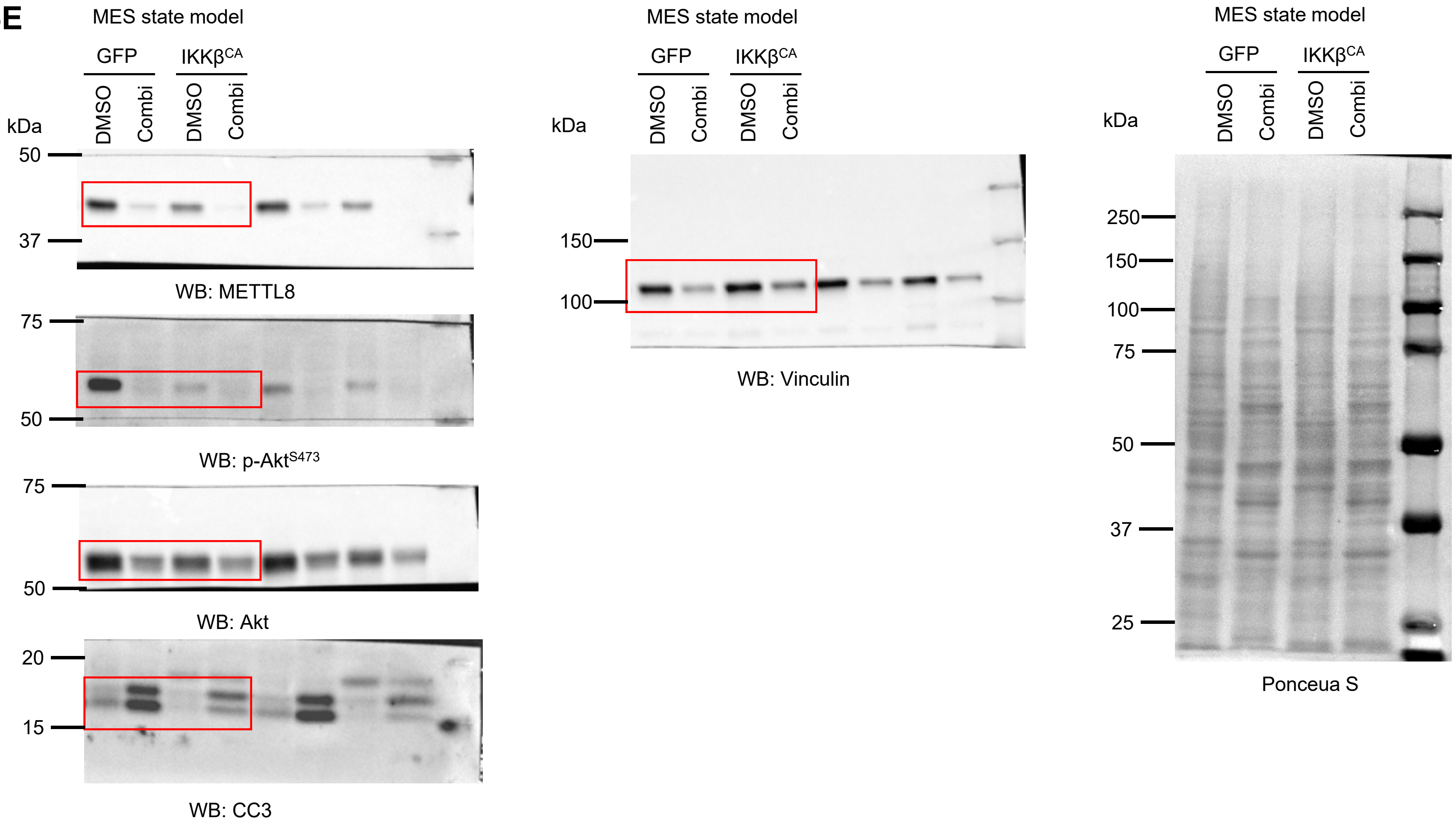

MES state model
GFP
IKKβCA
DMSO
Combi
DMSO
Combi
kDa
50
37
WB: METTL8
75
50
WB: p-AktS473
75
50
WB: Akt
20
15
WB: CC3
MES state model
GFP
IKKβCA
DMSO
Combi
DMSO
Combi
kDa
150
100
WB: Vinculin
MES state model
GFP
IKKβCA
DMSO
Combi
DMSO
Combi
kDa
250
150
100
75
50
37
25
Ponceua S

Fig. 6F

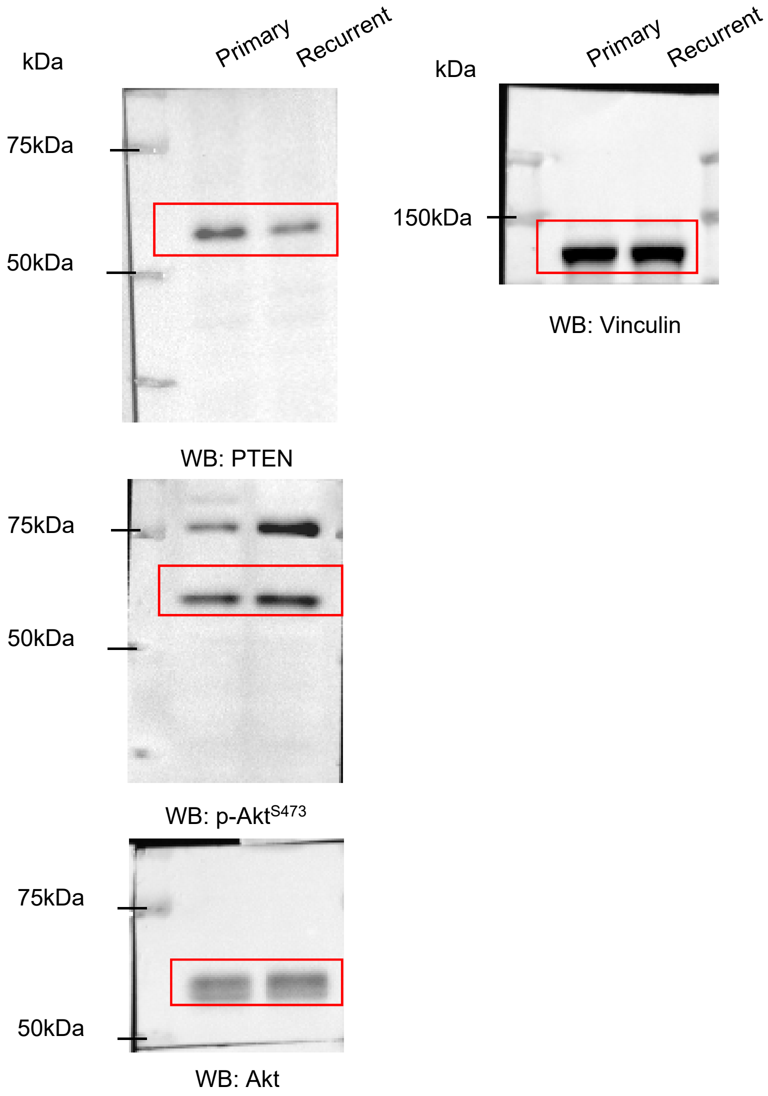
kDa
Primary
Recurrent
75kDa
50kDa
WB: PTEN
75kDa
50kDa
WB: p-Akt$^{S473}$
75kDa
50kDa
WB: Akt
kDa
Primary
Recurrent
150kDa
WB: Vinculin
